# Hypoxia regulates the mitochondrial activity of hepatocellular carcinoma cells through HIF/HEY1/PINK1 pathway

**DOI:** 10.1038/s41419-019-2155-3

**Published:** 2019-12-09

**Authors:** David Kung-Chun Chiu, Aki Pui-Wah Tse, Cheuk-Ting Law, Iris Ming-Jing Xu, Derek Lee, Mengnuo Chen, Robin Kit-Ho Lai, Vincent Wai-Hin Yuen, Jacinth Wing-Sum Cheu, Daniel Wai-Hung Ho, Chun-Ming Wong, Huafeng Zhang, Irene Oi-Lin Ng, Carmen Chak-Lui Wong

**Affiliations:** 1 Department of Pathology, The University of Hong Kong, Hong Kong, China; 2 State Key Laboratory for Liver Research, The University of Hong Kong, Hong Kong, China; 3 School of Life Sciences, University of Science and Technology of China, Hefei, China

**Keywords:** Cancer metabolism, Stress signalling

## Abstract

Hypoxia is commonly found in cancers. Hypoxia, due to the lack of oxygen (O_2_) as the electron recipient, causes inefficient electron transfer through the electron transport chain at the mitochondria leading to accumulation of reactive oxygen species (ROS) which could create irreversible cellular damages. Through hypoxia-inducible factor 1 (HIF-1) which elicits various molecular events, cells are able to overcome low O_2_. Knowledge about the new molecular mechanisms governed by HIF-1 is important for new therapeutic interventions targeting hypoxic tumors. Using hepatocellular carcinoma (HCC) as a model, we revealed that the HIF-1 and the Notch signaling pathways cross-talk to control mitochondrial biogenesis of cancer cells to maintain REDOX balance. From transcriptome sequencing, we found that HEY1, a transcriptional repressor, in the NOTCH pathway was consistently induced by hypoxia in HCC cell lines. We identified a strong hypoxia response element (HRE) in *HEY1* by chromatin immunoprecipitation (ChIP) and luciferase reporter assays. Transcriptome and ChIP sequencing further identified PINK1, a gene essential for mitochondrial biogenesis, as a novel transcriptional target of HEY1. HCC cells with HEY1 knockdown re-expressed PINK1. HEY1 and PINK1 expressions inversely correlated in human HCC samples. Overexpression of HEY1 and under-expression of PINK1 were detected in human HCC and associated with poor clinical outcomes. Functionally, we found that overexpression of HEY1 or knockdown of PINK1 consistently reduced mitochondrial cristae, mitochondrial mass, oxidative stress level, and increased HCC growth.

## Introduction

Hepatocellular carcinoma (HCC), the major form of primary liver cancer derived from hepatocytes, ranks the second most fatal malignancy in Mainland China and Hong Kong. High death rate in HCC patients is mainly due to late symptom presentation and the lack of curative therapy. Surgical resection and liver transplantation remain the most effective treatments which are only eligible for a minority of HCC patients as the majority of patients suffer from poor liver functions or metastasis. Currently, there are two FDA-approved first line drug for advanced HCC patients, Sorafenib and Lenvatinib, which can prolong the survival of HCC patients for <3 months^1,2^. Regorafenib and Nivolumab were recently approved by FDA as second-line treatments^3,4^. Median life expectancy of HCC patients is <2 years. Better understanding of the molecular mechanisms and the subsequent metabolic derangements of HCC will open new opportunities for more efficient HCC therapeutic interventions.

Hypoxia, oxygen (O_2_) deprivation, is frequently found in regions of HCC that are devoid of functional blood vessels. Hypoxia can also be induced during palliative HCC treatment such as transcatheter arterial (chemo) embolization (TAE/TACE) with the initial principle to restrict tumor growth through blockade of blood supply^5^. In addition, rapidly proliferating HCC cells quickly consume therefore deplete O_2_ in the tumor microenvironment of HCC. The key molecular mechanism by which cells adapt to hypoxia is through transcription factor, hypoxia-inducible factors (HIF). HIF is a dimer composed of the O_2_ labile subunit, HIF-1/2α, and the constitutively expressed subunit, HIF-1β^6^. In the presence of O_2_, HIF-1/2α is hydroxylated by prolyl hydroxylases (PHDs), thereby allowing the recognition and binding of Von Hippel-Lindal (VHL) protein which mediates ubiquitin-proteosomal degradation of HIF-1/2α^7^. In the absence of O_2_, hypoxia-inducible factors (HIF)-1/2α escaped from degradation and is stabilized and dimerizes with HIF-1β, forming a transcription complex that binds to the DNA sequences containing the hypoxia response elements (HREs: -A/GCGTC-) to drive gene transcription^8^. Overexpression of HIF-1α and HIF-2α are invariably detected in HCC and is associated with poor clinical outcomes^5^.

HIF transcriptionally activates a wide repertoire of genes that promote tumor growth and metastasis. HIF-1 is particularly crucial for shifting the metabolic program of cancer cells from oxidative phosphorylation to glycolysis. In the presence of O_2_, cells convert glucose into pyruvate which enters the TCA cycle in the mitochondria. NADH produced from glycolysis and TCA cycle enters the electron transport chain (ETC) and acts as the electron source to initiate the electron transfer through different complexes of the ETC ultimately to electron acceptor, O_2_ to generate ATP, a process called oxidative phosphorylation. When O_2_ content is low, the electron flow in the ETC becomes imbalanced, generating a large amount of reactive oxygen species (ROS), which are harmful to cells by triggering cell cycle arrest and apoptosis. HIF-1 allows hypoxic cells to survive oxidative stress by generating ATP through glycolysis through induction of glucose transporter 1 (GLUT1)^9^, Hexokinase 2 (HK2)^10,11^, pyruvate dehydrogenase kinase 1 (PDK1)^12^, and lactate dehydrogenase (LDHA)^13,14^. GLUT1 facilitates the uptake of glucose which increases glycolytic rate. HK2 is the first step of glycolysis which phosphorylates glucose. PDK1 prevents the conversion of pyruvate to acetyl-CoA, thereby shunting pyruvate into glycolysis so that pyruvate could be converted into lactate by LDHA to generate ATP. While increasing glycolytic activity, HIF-1 decreases mitochondrial activity by inducing the less active ETC subunits in complex 1 and complex 4, NDUFA4L2 and COX4/2, to decelerate the electron transfer through the ETC therefore preventing the accumulation of ROS^15,16^. Furthermore, HIF-1 decreases mitochondrial biogenesis through its transcriptional target, MAX interactor 1 (MXI), which inhibits C-MYC-mediated transcription of peroxisome proliferator-activated receptor gamma, coactivator 1 beta (PGC-1β)^17^.

Similar to the HIF signaling pathway, the Notch signaling pathway, is a key regulator of gene expression. The Notch pathway plays an important role in embryonic development as it regulates many genes important for differentiation hence determination of cell fate. The Notch pathway is activated when the Notch receptor was bound by the ligands of the Delta or Jagged family, thereby inducing the cleavage of the Notch receptor and the release of the intracellular domain of the Notch receptor (NICD) to the nucleus^18^. The NCID then binds to the promoters of Hes Family BHLH Transcription Factor (HES) and Hes-Related Family BHLH Transcription Factor With YRPW Motif (HEY) genes to activate thee RBPJκ-driven transcription. HES (1–7) and HEY (1, 2, L) belong to the basic helix-loop-helix transcription factor family. Members in the HES and HEY families generally repress transcription by recruiting co-repressors to their target genes. HES and HEY bind to DNA sequences containing the Notch-responsive elements (NREs: CACNAG, CANNTG)^18^. Typical targets that HES and HEY families repress include MYOD1 and MASH which are crucial for myogenesis and neuronal differentiation, respectively. Given their repressive roles on differentiation, HES and HEY families are important for embryonic stem cell maintenance. HEY was also found to be involved in liver cancer stem cell maintenance^19^. Intriguingly, cross-talks between the Notch and the HIF signaling pathways were found in neural stem cells, myogenic stem cells, and embryonic carcinoma cells to control transcription. Active HIF-1α forms a complex with NICD to enhance the stability of NICD^20^. NICD in turn recruits HIF-1α to the NREs of HES1 and HEY2 to maintain stem cells in undifferentiated state^20^. Recently, chromatin immunoprecipitation sequencing (ChIP-seq) revealed a large number of potential transcriptional targets of HES and HEY families, suggesting novel roles of the HES and HEY families. Although increasing evidence confirmed that the cross-talks of the HIF and the NOTCH pathways exist, how these signaling pathways intersect and integrate to control the transcriptional program of cancer cells, especially in HCC, remains largely unexplored. Intriguingly, our data showed that HEY1 was consistently induced by hypoxia in all HCC cell lines and was over-expressed in human HCC. Overexpression of HEY1 was significantly associated with more advanced tumor stages and poor overall survival. By ChIP and luciferase reporter assay, we found that HIF-1 complex directly bound to the HREs of HEY1 to activate its transcription in HCC cells. Furthermore, our in-house transcriptome sequencing data revealed an unprecedentedly documented target of HEY1, PTEN Induced Putative Kinase 1 (PINK1), which is a mitochondrial serine/threonine kinase crucial for the generation of healthy mitochondria. We further demonstrated that HEY1 repressed transcription of PINK1 and their expressions were inversely correlated in human HCC patients. We showed that HEY1 repressed mitochondrial biogenesis and ROS accumulation. In line with our expression studies, knockdown of HEY1 repressed tumor growth whereas knockdown of PINK1 augmented tumor growth in vivo. Taken together, we have revealed a novel molecular mechanism by which HIF-1 reduced oxidative stress through HEY1/PINK1, providing HCC cells survival advantages.

## Materials and methods

### Patient samples and cell lines

Human HCC and their paired non-tumorous liver tissues were surgically resected at Queen Mary Hospital. Use of human samples was approved by the Institutional Review Board of the University of Hong Kong/ Hospital Authority Hong Kong West Cluster. The patients signed consent forms to acknowledge the use of their resected tissues for research purposes. Human HCC cell line MHCC97L was a gift from Fudan University (Dr. Z.Y. Tang) of Shanghai. HeLa, PLC/PRF/5, HepG2, and Hep3B were purchased from American Type Culture Collection. Huh7 was obtained from Prof H. Nakabayshi from Hokkaido University School of Medicine, Japan. Human HCC cell line CLC1, CLC2, CLC11, CLC13 were gifts from Prof. Lijian Hui at Shanghai Institute of Biochemistry and Cell Biology^21^. All cell lines were cultured in Dulbecco’s Modified Eagle Medium (DMEM) supplemented with 10% feta bovine serum. Cell cultures were authenticated and checked routinely to be mycoplasma-free. Use of human specimens was approved by Institutional Review Board (IRB). Consent from patients was obtained. All cell lines were authenticated by STR profiling and tested as mycoplasma-free. Sample size of human HCC samples was chosen based on G-power calculation.

### Establishment of knockdown, knockout and overexpression HCC subclones

For the establishment of different knockdown cells, shRNA sequences targeting HEY1, PINK1, HIF-1α, HIF-2α, non-target control (NTC) were inserted into pLKO.1-puro vector by AgeI digestion^22^. pLKO (Sigma) plasmids were transfected into cells by lenti-viral approach and underwent puromycin selection for 7–14 days. HIF-1α stable KO HCC cell lines were established and validated by TALEN approach, as we previously described^23^. For the establishment of HEY1 over-expressing cells, we first inserted the open reading frame of HEY1 into pLenti6 (Thermo) vector by BamHI and XhoI digestion. pLenti6-HEY1 construct was transfected into cells by lenti-viral approach and underwent blasticidin selection for 14 days. For the establishment of PINK1 over-expressing system, we employed the CRISPR-dCas9 synergistic activator system (SAM)^24^. We sequentially and stably transfected dCas9-VP64, MS2-p65-HSF1, and sgRNAs targeting the promoter of PINK1 and HEY1 into MHCC97L cells, by lenti-viral approach as described^24^. ShRNA and sgRNA sequences are provided in Supplementary table 1.

### Transcriptome sequencing

Transcriptome sequencing was performed in Huh7-NTC, -shHEY1-17 cells that were exposed to 1% O_2_ for 24 h. PolyA + mRNA library was prepared with TruSeq standard mRNA sample Prep kit (Illumina). In total 100 bp paired-end sequencing (Axeq Technologies) was performed by Illumina HiSeq2000. All data were analyzed by TopHat-Cufflinks pipeline^25^. Transcriptome sequencing data for Huh7 cells were deposited with Bioproject accession number PRJNA574563. Transcriptome sequencing data on HCC tissues were previously deposited with Bioproject accession number PRJNA294031.

### Luciferase reporter assay

WT and Mut HEY1 HREs were inserted into pGL2 luciferase reporter by BgIII digestion. Huh7 and PLC cells were transfected with pGL2-HEY1-WT and –Mut plasmids together with Renilla plasmid at 200 to 1 ratio. After one overnight, cells were exposed to 20 and 1% O_2_ for 24 h and Dual-Luciferase® Reporter assay (Promega) was performed following manufacturer’s protocol.

### Quantitative real-time PCR (qRT-PCR)

Total RNA extraction was performed with TRIzol (Sigma). Reverse transcription was performed with GeneAmp Gold RNA PCR Core Kit (Applied Biosystems). qRT-PCRs of HEY1, PINK1, the internal normalization controls 18 S or HPRT were performed using the Taqman® Gene Expression Assay in human tissue sampmles while qRT-PCRs of these genes in cell lines were performed with SYBR Green qPCR Master Mix (Applied Biosystems) with specific primers provided in Supplementary table 2.

### ChIP assay

MHCC-97L cells were fixed formaldehyde, lysed with SDS buffer, and sonicated. Sheared DNA was blocked with salmon sperm DNA/protein A agarose slurry (Merck Millipore) and immuno-precipitated with HIF-1α (Abcam; ab1), HIF-1β (Abcam; ab2), V5 (Abcam; ab15828), and IgG antibodies (Invitrogen; 10500 C and Santa Cruz; sc-2762). Antibody/protein/DNA complex was incubated with agarose beads and washed with low-salt buffer, high-salt buffer, and LiCl wash buffer according to manufacturer’s protocol (Millipore). DNA elution was done with 1% SDS/0.1 M NaHCO_3_. Eluted DNA was de-cross-linked with 0.2 M NaCl at 65 °C overnight. DNA was extracted by phenol-chloroform (Sigma) followed by qRT-PCR with SYBR Green qPCR Master Mix with specific primers provided in Supplementary table 2.

### Clinicopathological correlation

The mRNA level of HEY1 and PINK1 in HCC patients was correlated with different clinicopathological parameters in HCC patients by SPSS20.0 software (SPSS, Inc.)^26^. Briefly, the parameters were analyzed by pathologist upon surgical resection. The clinicopathological parameters included venous invasion, direct liver invasion, tumor size, absence of tumor encapsulation, presence of tumor microsatellite formation, cellular differentiation by Edmondson grading, and association with hepatitis B infection. Clinicopathological correlations of HEY1 and PINK1 expression were performed with Fisher Exact test. Survival test was done by Kaplan–Meier curve and log-rank test. *P* < 0.05 was considered statistically significant.

### TCGA data

The TCGA data are available in the cBioPortal for Cancer Genomics website http://www.cbioportal.org/.

### Animal experiments

For orthotopic implantation, 1 × 10^6^ luciferase-labelled MHCC97L cells were injected into left lobes of the livers of 5–7-week-old male BALB/c nude mice while 3 × 10^6^ Hepa1-6 cells of C57BL/6 mice. Mice were administered with 100 mg/kg D-luciferin (Caliper) via intraperitoneal (i.p.) injection prior to bioluminescent imaging using Xenogen IVISTM100 Imaging System. For subcutaneous injection, 5–7-week-old male BALB/c nude mice were subcutaneously injected with 1 × 10^6^ HCC cells at their right flanks. Three dimensions of the tumors were measured by caliper and tumor volume was calculated the following formula: width × length × height × 0.52 (mm^3^). All animal studies were approved by the Committee on the Use of Live Animals in Teaching and Research, the University of Hong Kong and performed under the Animals (Control of Experiments) Ordinance of Hong Kong. No specific randomization method was used. Sample size of animals was chosen based on significant *p* values.

### Metabolic assays

Cells were stained with 10 µM 10-N-Nonyl acridine orange (NAO) (Thermo Fisher) in 0.1% bovine serum albumin (BSA)/ phosphate-buffered saline (PBS) for 15 min followed by flow cytometry analysis with BD FACSCantoII Analyzer (BD Biosciences) and FlowJo software (FlowJo). Cells were stained with 10 µM CM-H_2_DCFDA (Thermo Fisher) in PBS for 10 min followed by flow cytometry analysis as abovementioned.

### Cell proliferation assay

In total 1 × 10^4^ HCC cells were seeded onto each well of 12-well plates. Cells were exposed to 20 and 1% O_2_ conditions at the given time point. Media were replenished and cell number was evaluated by automated cell counter daily.

### Electron microscopy

In total 1 × 10^6^ cells seeded on TC plates were fixed with ice 4% formalin for one overnight at 4^o^C. Cells were scraped off, centrifuged at low speed, and stored in 1.5 mL 4% formalin. Formalin was replaced with 0.2 M sucrose for overnight. Cells were fixed with 1% OsO_4_ for 1 h. Cells were rinsed and dehydrated with gradient of EtOH and placed in EMBed 812: proylene oxide overnight in desiccator. Cells were then embedded in Beam capsules and baked in oven at 60 °C oven for 48 h. Cells were sectioned 0.5 µm thick and collected on grids. Grids were stained with uranyl acetate for 15 min and then lead citrate for 5 min. Cells were imaged with Philips CM100 transmission electron microscope.

### Antibodies

The antibodies against HEY1 (LifeSpan BioSciences; LS-C107603), HEY1 (abcam, ab22614), HIF-1α (Cell Signaling; #3716 S), HIF-2α (abcam; ab199), PINK1 (Cell Signaling; #6946 S), Histone H3 (Millipore; 05–928), and β-actin (Sigma; A5316) were used for Western blotting.

### Statistical methods

Exact sample size (N) for each experimental condition is indicated in figure legend of each experiment. Data represent technical repeats for in vitro experiment and all experiments have been repeated three times with consistent trends. Data are presented as biological repeats for in vivo experiment in different animals. Student’s *t*-test for comparison of two groups or one-way ANOVA, Turkey’s multiple comparisons for multiple groups are used in different experiments as indicated in figure legend. All tests are two-sided. P values for different comparisons are indicated in figure legend. Means are the center values and error bars represent standard deviations. Data point is excluded if it deviates from mean with more than 3 standard deviations. Investigators were not blinded to the group allocation during experiment and when assessing the outcome in all experiments including animal experiments. There is no estimate of variation within each group of data. Variance is similar between the groups that are being statistically compared.

## Results

### HEY1 as the only family member induced by hypoxia in HCC

**Fig. 1:**
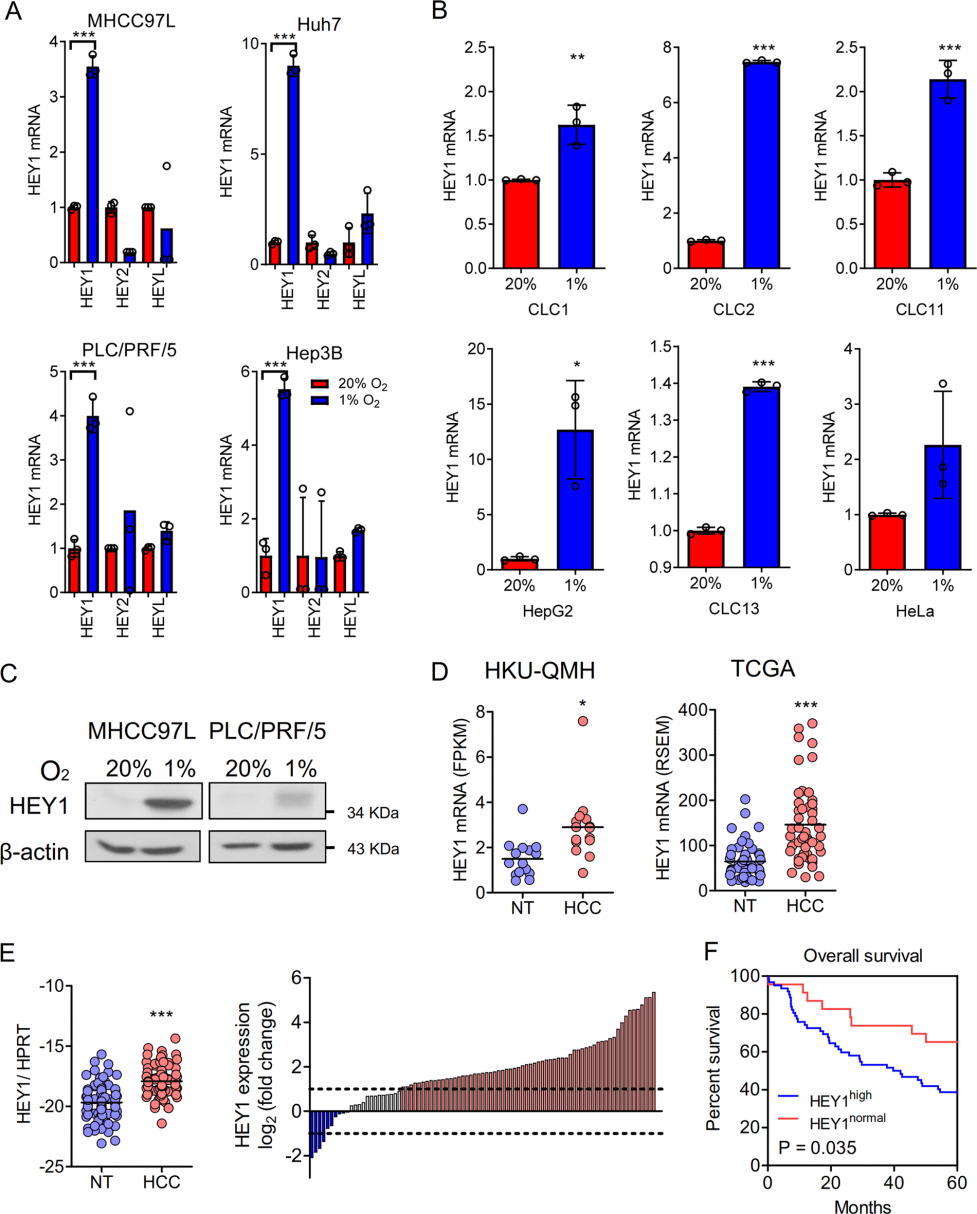
Expressions of HEY family members in human HCC cell lines and clinical samples. **a** RT-qPCR of HEY1, HEY2, HEYL mRNA expression levels in 4 human HCC cell lines (MHCC97L, Huh7, PLC/PRF/5, Hep3B) exposed to 20 and 1% O_2_ for 24 h. **b** RT-qPCR confirmed HEY1 mRNA expressions in 5 human HCC cell lines (CLC1, CLC2, CLC11, HepG2, CLC13) and 1 human cervical cancer cell line (HeLa). Data were normalized to the corresponding values in 20% O_2_ and house keeping gene, 18 S. **c** HEY1 protein expression in MHCC97L and PLC/PRF/5 exposed to 20 and 1% O_2_ for 48 h. **d** HEY1 mRNA expression in (middle) in-house 16 pairs and (right) 49 pairs from TCGA database of human HCC tissues and corresponding non-tumorous liver tissues (NT) was detected by transcriptome sequencing. **e** Left: HEY1 mRNA expression was determined by qRT-PCR in 87 cases of in-house (HKU-QMH) human HCC and NT tissues. Right: Waterfall plot shows that HEY1 was over-expressed in 72.41% (63/87) of HCC patients by at least 2 fold. **f** HEY1 overexpression ( ≥ 2 fold) was closely associated with poor overall survival in HCC patients. Data are presented as mean ± s.d. (Student’s *t*-test, *P < 0.05, **P < 0.01, ***P < 0.001).

To study the common HIF transcriptional targets that might implicate in HCC, we performed RT-qPCR in 4 human HCC cell lines, MHCC97L, Huh7, PLC, and Hep3B, that were exposed to hypoxia (1% O_2_) and normoxia (1% O_2_) for 24 h. We found that HEY1 was consistently and significantly induced in all human HCC cell lines (Fig. 1a). However, expression values of two other family members in the HEY family, HEY2 and HEYL, were not induced significantly by hypoxia (Fig. 1a). We further validated our finding by qRT-PCR in additional HCC cell lines including CLC1, CLC2, CLC11, HepG2, and CLC13 and found that HEY1 was consistently induced by hypoxia in the abovementioned HCC cell lines (Fig. 1b). HEY1 was also induced by hypoxia in a cervical cancer cell line, HeLa, suggesting that hypoxia-induced HEY1 expression is not limited to HCC but it’s universal in other cancer types (Fig. 1b). The induction of HEY1 protein was further confirmed in selected HCC cell lines (Fig. 1c).

### HEY1 is over-expressed in HCC

Transcriptome sequencing data in a discovery in-house cohort consisting of 16 cases of HCC and their corresponding non-tumorous (NT) tissues and TCGA database revealed that HEY1 was over-expressed (Fig. 1d). qRT-PCR further confirmed that HEY1 was profoundly over-expressed in an expanded in-house cohort of 87 HCC patients (Fig. 1e). In total 63/87 (72.4%) of HCC patients displayed increased HEY1 mRNA expression by at least 2-fold in their HCC tissues relative to NT tissues (Fig. 1e). HEY1 overexpression was also closely correlated with shorter overall survival (Fig. 1f) and more advanced tumor stages (Supplementary Table S3) in HCC patients.

### HEY1 is a transcriptional target of HIFs

**Fig. 2:**
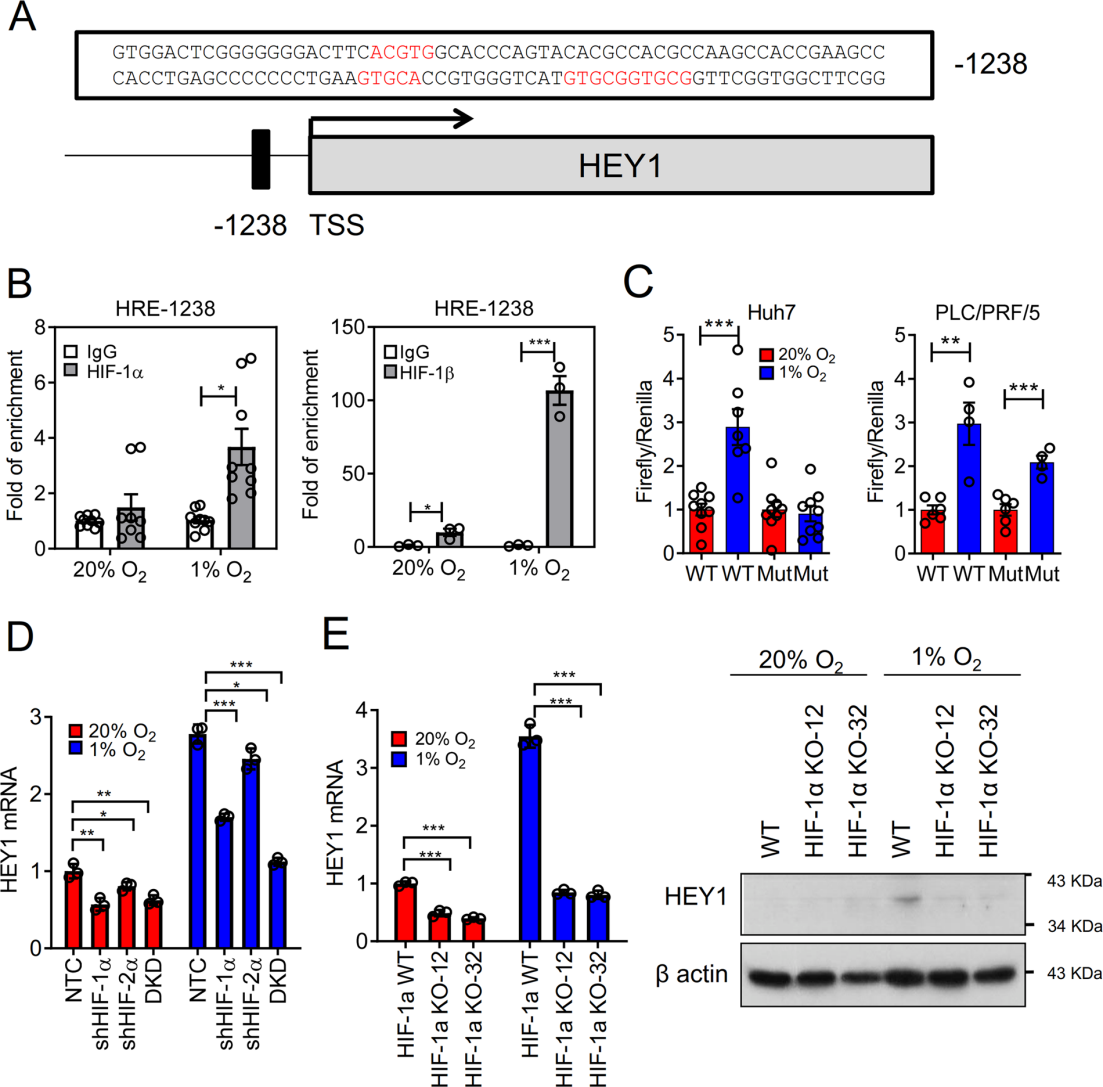
HEY1 is a transcriptional target of HIF-1. **a** 4 copies of HREs (red) were found in 1238 bp promixal to transcription start site (TSS) of HEY1. **b** ChIP assay was performed with HIF-1α, HIF-1β, and IgG control antibodies in MHCC97L cells exposed to 20 and 1% O_2_ for 24 h. **c** Huh7 and PLC/PRF/5 cells were transfected with p2.1 Firefly luciferase reporter encompassing wildtype (WT) or mutated (Mut) HEY1 HREs and Renilla luciferase control plasmids. Transfected cells were exposed to 20 and 1% O_2_ for 24 h for dual luciferase activities quantification. **d** HEY1 mRNA expression in MHCC97L-shNTC, -shHIF-1α, -shHIF-2α, and HIF-1α and HIF-2α double knockdown (DKD) subclones exposed to 20 and 1% O_2_ for 24 h. **e** HEY1 mRNA (left) and protein (right) expressions in MHCC97L-WT and -HIF-1αKO subclones (established by TALEN) exposed to 20 and 1% O_2_ for 24 (mRNA) and 48 (protein) hours. For qRT-PCR, values were normalized to house keeping gene, 18 S. Data are presented as mean ± s.d. (Student’s *t*-test, *P < 0.05, **P < 0.01, ***P < 0.001).

Next, we asked if HEY1 is regulated by HIFs. We found 4 copies of the putative hypoxia response element (HRE) encompassing consensus sequence –A/GCGTG- near the promoter region of *HEY1* (Fig. 2a). Chromatin immunoprecipitation (ChIP) assay clearly showed that HIF-1α and HIF-1β bound to the putative HRE of HEY1 as indicated by the significant fold of enrichment as compared to IgG control in MHCC97L that were exposed to 1% O_2_ for 24 h (Fig. 2b). To study whether HIF-1 functionally activates the HRE, we cloned the wildtype (WT) and mutated (Mut) HREs of *HEY1* in front of luciferase promoter. We found that hypoxia significantly induced the luciferase activity of the WT HRE of *HEY1* in two HCC cell lines, Huh7 and PLC. In contrast, hypoxia did not induce the luciferase activity of the Mut HRE of *HEY1* as much as the WT HRE of *HEY1* (Fig. 2c). We have established HIF knockdown and knockout stable clones in MHCC97L cells (Fig. S1A-B). Importantly, we found that hypoxia-induced HEY1 expression could be abrogated greatly when we knocked down HIF-1α and abrogated mildly when we knocked down HIF-2α in MHCC97L cells (Fig. 2d). Hypoxia-induced HEY1 expression was the most suppressed in MHCC97L cells with HIF-1α and HIF-2α double knockdown (DKD) as compared to single knockdown of either gene. Genetic knockout of HIF-1α in MHCC97L cells using transcription activator-like effector nuclease (TALEN) approach consistently abrogated hypoxia-induced HEY1 mRNA and protein expressions (Figs. S1C-D and 2E). All these data together showed that HEY1 is transcriptionally activated by HIFs.

### HEY1 transcriptionally represses mitochondrial protein PINK1

HEY1 is a transcriptional repressor and to explore novel targets and functions of HEY1, we knocked down HEY1 in Huh7 cells (Fig. S2) and performed transcriptome sequencing in Huh7 cells that were exposed to 1% O_2_. We focused on the genes that were re-expressed upon knockdown of HEY1. 2217 genes expressed at least 1.2 fold higher in HEY1 knockdown cells relative to control cells with FPKM values ≥ 1. We also retrieved the only available and published ChIP sequencing data on HEY1 in mammalian system and selected the top 150 genes which showed enrichment in the ChIP^27^. Combining our own transcriptome sequencing dataset with the ChIP sequencing dataset published by others, we found 12 genes that are commonly regulated by HEY1 (Supplementary Table S4).

**Fig. 3:**
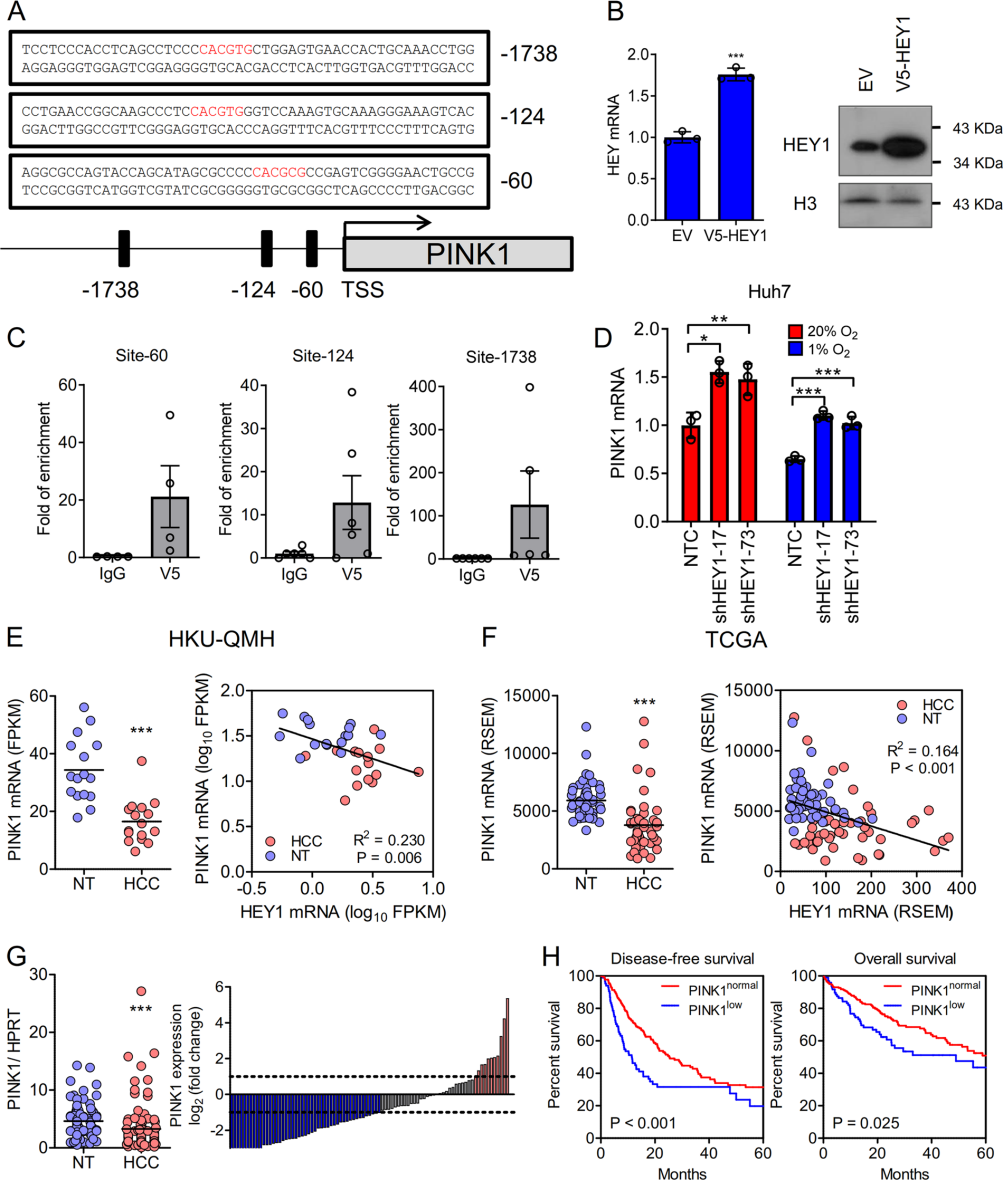
HEY1 represses PINK1. **a** 3 putative E boxes (red) were located upstream to the TSS of PINK1. **b**, **c** ChIP assay was performed in Hey1 over-expressing HCC cells (Huh7-V5-HEY1) with V5 and IgG control antibodies. **d** PINK1 mRNA expression in Huh7-NTC, -shHEY1-17, shHEY-73 cells that were exposed to 20 and 1% O_2_ for 24 and 48 h, respectively. **e** Left: transcriptome sequencing revealed that PINK1 was down-regulated in human HCC. Right: PINK1 and HEY1 expressions were inversely correlated in our in-house 16 cases of HCC and NT tissues. **f** Left: PINK1 mRNA expression in HCC and NT tissues in 49 HCC patients from the TCGA database. Right: PINK1 and HEY1 expressions were inversely correlated in HCC and NT tissues from TCGA data base. **g** Left: RT-qPCR confirmed that PINK1 was down-regulated in an expanded cohort of 87 HCC patients. Waterfall plot shows that PINK1 was under-expressed in 47/87 (54.02%) HCC patients by at least 1 fold. **h** PINK1 was defined as down-regulated with Z-score < -1 otherwise normal. PINK1 down-regulation was associated with shorter disease-free and overall survivals in HCC patients. RSEM: RNA-Seq by Expectation-Maximization. For qRT-PCR, values were normalized to house keeping gene, 18 S or HPRT. Data are presented as mean ± s.d. (Student’s *t*-test, *P < 0.05, **P < 0.01, ***P < 0.001).

Among these genes, we were particularly attracted by PINK1 given its critical roles in mitochondrial biogenesis. We identified three putative Notch-responsive elements, where HEY1 might bind, near the promoter of PINK1 (Fig. 3a). We generated V5-tagged HEY1 over-expressing Huh7 cells and performed ChIP assay with V5 antibody (Fig. 3b). V5 antibody successfully immunoprecipitated the three putative HEY1-binding sites in PINK1 (Fig. 3c). We further confirmed that knockdown of HEY1 significantly elevated PINK1 expression (Fig. 3d). More strikingly, linear regression model showed that HEY1 and PINK1 mRNA expressions were reversely correlated in our in-house transcriptome sequence database of 16 pairs HCC and NT tissues as well as TCGA data base of 49 pairs HCC and NT tissues (Fig. 3e, f). Consistent with our hypothesis, PINK1 down-regulation was frequently found in HCC patients (Fig. 3f, g). PINK1 down-regulation was also significantly associated with poor survival and poor cellular differentiation in HCC patients (Fig. 3h and Supplementary Table S5). HEY1 overexpression, PINK1 down-regulation, and their correlation were also observed in intrahepatic cholangiocarcinoma (ICC), another type of primary liver cancer which is derived from bile duct (Supplementary Fig. S3A-C). We next asked if this trend could also be observed in renal cell carcinoma, a malignancy which frequently harbors VHL mutations leading to constitutive activation of HIF-1α. Interestingly, we found that HEY1 was over-expressed in the tumorous tissues as compared to non-tumorous kidney tissues while PINK1 was under-expressed (Supplementary Fig. S3D-E). More strikingly, linear regression model showed that HEY1 and PINK1 mRNA expressions were reversely correlated in 72 cases of kidney cancer and non-tumorous tissues (Supplementary Fig. S3F), suggesting that our findings are not restricted to liver cancers.

### HEY1 represses PINK1 to alleviate mitochondrial reactive oxygen species (ROS) production

**Fig. 4:**
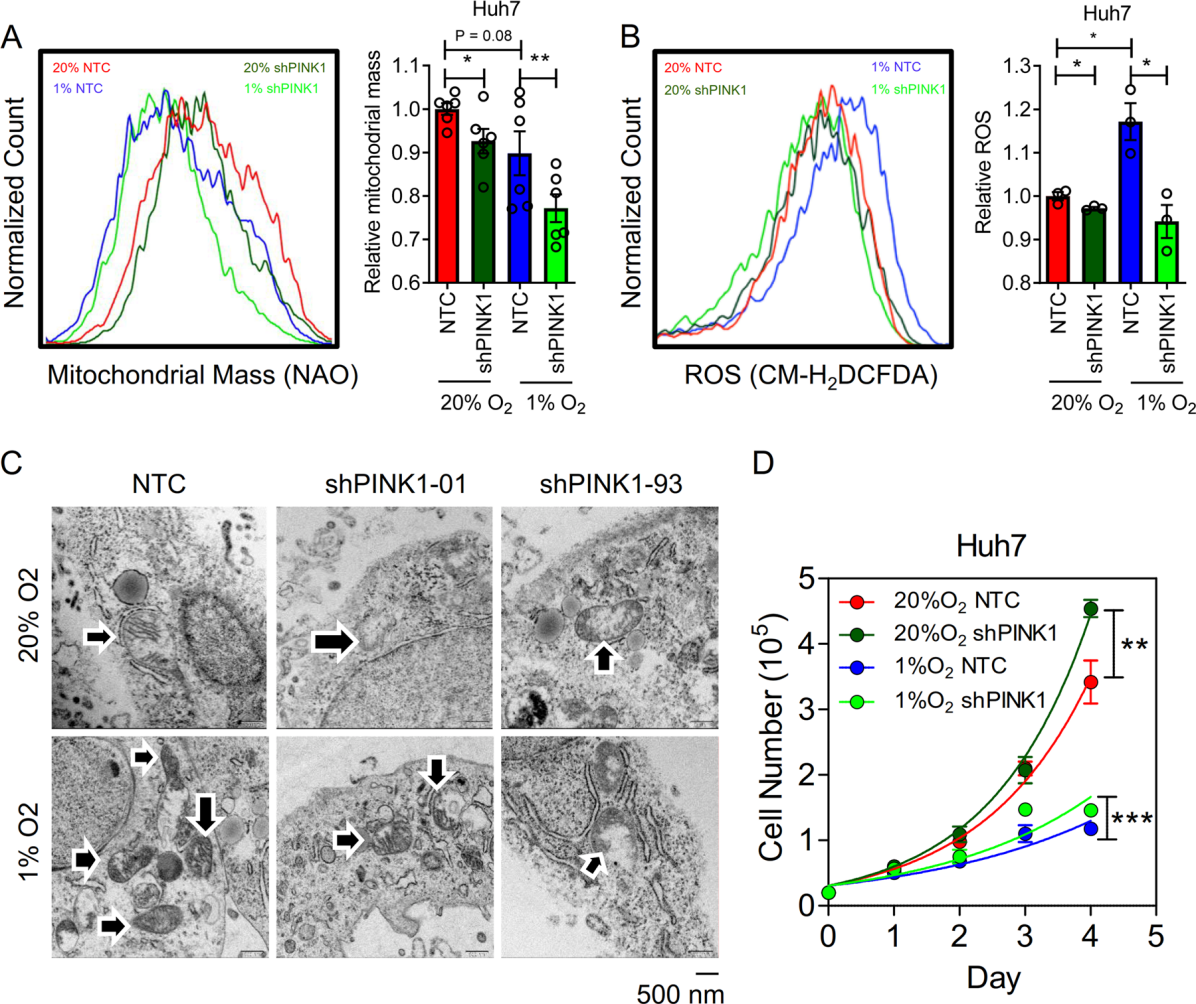
PINK1 increases mitochondrial mass and ROS and inhibits HCC proliferation. **a** Mitochondrial mass, **b** ROS in Huh7-NTC and shPINK1 subclones exposed to 20 and 1% O_2_ for 24 h. **c** Huh7-NTC, -shPINK1-01, shPINK1-93 cells were exposed to 20 and 1% O_2_ for 24 h. Cell images under transmission electron microscope (TEM). **d** Proliferation of Huh7-NTC and shPINK1 subclones exposed to 20 and 1% O_2_ for 4 days. Data are presented as mean ± s.d. (Student’s *t*-test, *P < 0.05, **P < 0.01, ***P < 0.001).

PINK1 plays a critical role in mitochondrial biogenesis. Mitochondria is a major site where reactive oxygen species (ROS) are produced. Cancer cells actively generate ROS due to mitochondrial mutations and hypoxia. When ROS production exceeds elimination, ROS create irreversible cellular damages which ultimately kill cancer cells. We speculated HEY1 might reduce mitochondrial biogenesis, thereby alleviating ROS level through repressing PINK1 expression. To confirm that PINK1 is playing a role in mitochondrial biogenesis in HCC cells, we established in Huh7-NTC and -shPINK1 cells (Supplementary Fig. S4) and performed Nonyl Acridine Orange (NAO) staining to evaluate the mitochondrial mass in 20 and 1% O_2_ conditions. We found that mitochondrial mass was significantly reduced in HCC cells upon knockdown of PINK1 (Fig. 4a). To confirm the roles of PINK1 in mitochondrial biogenesis, we established PINK1 over-expressing (OE) Huh7 cells using CRISPR/dCas9-VP64 Synergistic Activation Mediator (SAM) system. In the SAM system, VP64 transcription factor (dCas9-VP64) and transcription co-activators (MS2-p65-HSF1) were guided to the promoter of PINK1 by sgRNA to activate endogenous PINK1 transcription. The effect of PINK-OE system on mitochondrial mass was not as obvious as shown in PINK-knockdown system. Although not reaching statistical significance, we found a trend that PINK1-OE cells only showed a marginal increase of mitochondrial mass only in 1% O_2_ condition (Supplementary Fig. S5). As ROS is produced in mitochondria, we hypothesized that ROS level would be reduced as mitochondrial mass is decreased. We stained the Huh7-NTC and –shPINK1 cells with a ROS dye, CMH_2_DCFDA. ROS level was reduced in PINK1-knockdown clones (Fig. 4b). To confirm the effects of PINK1 in mitochondrial structures, we imaged the Huh-NTC and –shPINK1 cells which were exposed to 20 and 1% O_2_ by transmission electron microscope (TEM). Under normoxic condition, cristae could be clearly observed in Huh7-NTC cells (Fig. 4c). On the contrary, cristae could not be properly formed in Huh7-shPINK1 cells. Interestingly, under hypoxic condition, mitochondrial number slightly increased while cristae remained intact in Huh7-NTC cells (Fig. 4c). However, the mitochondria were smaller in size as compared to normoxic control. Consistently, cristae could not be properly formed in Huh7-shPINK1 cells under hypoxic condition (Fig. 4c). Number of mitochondria was reduced in PINK1-knockdown HCC cells. The increase of mitochondria number and decrease of mitochondria size under hypoxia might be indicative of mitochondrial fission in hypoxia. Mitochondria undergo fusion (merging) and fission (division) to adapt to energetic requirements under different nutrient environmental and nutritional conditions. Mitochondria fusion increases mitochondria activity while mitochondrial fission reduces mitochondria activity and enhances glycolysis. It is reasonable that mitochondrial fission is observed in hypoxic cells as hypoxia switches metabolic mode to glycolysis. Our data showed that PINK1 is responsible for mitochondrial cristae formation and might be associated with mitochondrial fission. Interestingly, knockdown of PINK1 modestly increased HCC cell proliferation rate (Fig. 4d), suggesting that PINK1 acts as a growth repressor in HCC.

**Fig. 5:**
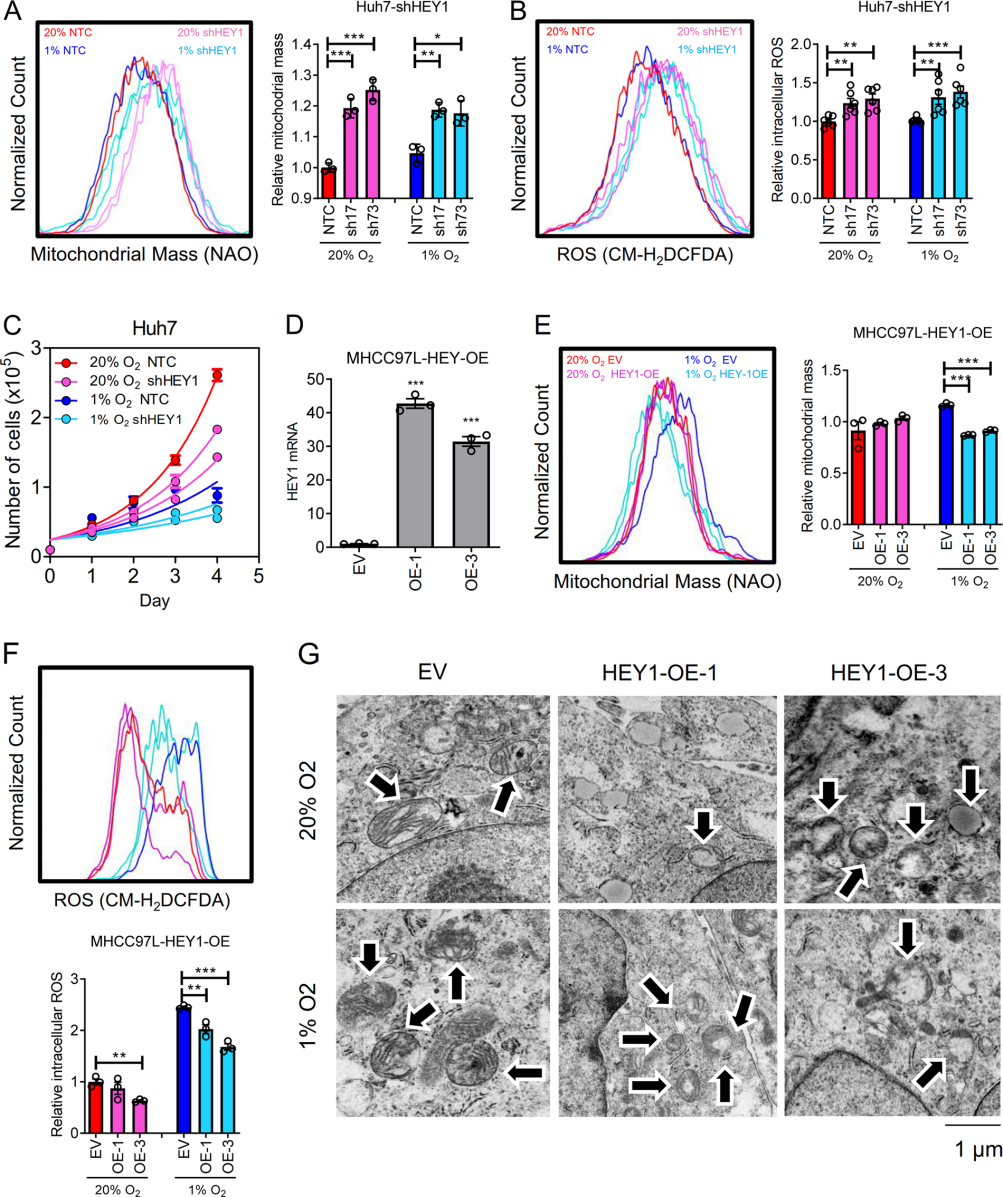
HEY1 decreases mitochondrial mass and ROS and promotes HCC proliferation. **a** Mitochondrial mass **b** ROS in Huh7-NTC and shHEY1 subclones exposed to 20 and 1% O_2_ for 24 h. **c** Proliferation of Huh7-NTC and shHEY1 subclones exposed to 20 and 1% O_2_ for 4 days. **d** HEY1 mRNA expression in MHCC97L-HEY1-OE cells established by CRISPR-dCas9-SAM system. **e** Mitochondrial mass and **f** ROS in MHCC97L-EV and –HEY1-OE subclones exposed to 20 and 1% O_2_ for 24 h. **g** Cell images of HEY1-OE cells under TEM. Arrows point to mitochondria. Data are presented as mean ± s.d. (Student’s *t*-test, *P < 0.05, **P < 0.01, ***P < 0.001).

Similarly, we performed NAO and CM-H_2_DCFDA staining in Huh7-NTC and –shHEY cells. Knockdown of HEY1 increased mitochondrial mass and ROS and decreased HCC cell proliferation rate (Fig. 5a–c). TEM showed that mitochondrial cristae were intact in the HEY1 knockdown cells as PINK1 expression was elevated in HEY1 knockdown cells (Supplementary Fig. S4). Reversely, HEY1 over-expressing (OE) MHCC97L cells established by CRISPR/dCas9-Vp64 SAM system (Fig. 5d) exhibited reduced mitochondrial mass and ROS (Fig. 5e, f). Resembling PINK1 knockdown, mitochondrial cristae could not be formed in HEY1-OE clones (Fig. 5g), suggesting a role of HEY1 in mitochondrial structures.

### In vivo effects of HEY1/PINK1 pathway in HCC growth

**Fig. 6:**
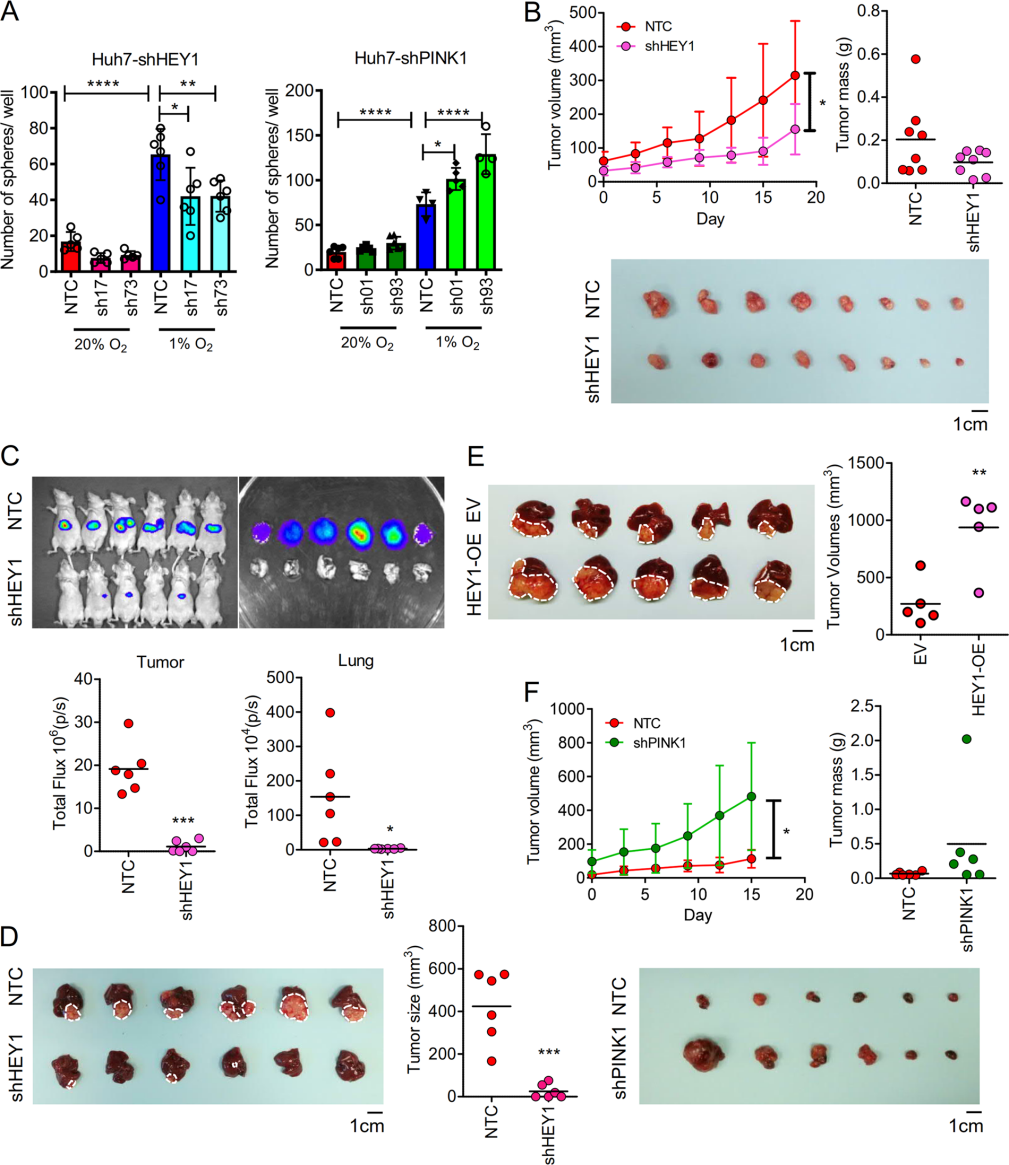
HEY1 promotes whereas PINK1 represses HCC growth in vivo. **a** Sphere formation assay. The number of spheres derived from Huh7-NTC and –shHEY1/PINK1 cells exposed to 20 and 1% O_2_ were counted. **b** In total 1 × 10^6^ Huh7-NTC and -shHEY1-17 cells were injected into BALB/c nude mice subcutaneously. Tumor volume was calculated based on the 3 dimensions of the tumors measured by caliper and tumor mass was measured when mice were sacrificed at day 18. In total 1 × 10^6^ luciferase-labelled MHCC97L-NTC and -shHEY1-17 cells were injected into the left lobes of the livers of BALB/c nude mice. (*n* = 8 for each group). **c** Bioluminescence of (left) tumors and (right) lung metastases in mice after 6 weeks of orthotopic implantation. **d** Livers were harvested 6 weeks post-injection and tumor size was measured. (*n* = 6 for each group). **e** 2 × 10^6^ MHCC97L-EV and -HEY1-OE cells were injected into BALB/c nude mice orthotopically. Livers were harvested 6 weeks post-injection and tumor size was measured. (*n* = 6 for each group). **f** 1 × 10^6^ Huh7-NTC and –shPINK1 cells were injected into BALB/c nude mice subcutaneously. Tumor volume was calculated based on the 3 dimensions of the tumors measured by caliper and tumor mass was measured when mice were killed at day 15. (*n* = 6 for each group) Data are presented as mean ± s.d. (one-way ANOVA, Turkey’s multiple comparison test in A for comparison of multiple groups, Student’s *t*-test in **b**, **c**, **e**, **f** for comparison between two groups, *P < 0.05, **P < 0.01, ***P < 0.001, ***P < 0.0001).

Hypoxia promoted the sphere formation ability in HCC while it was abrogated upon HEY-knockdown (Fig. 6a). Reversely, knockdown of PINK1 further promoted sphere formation (Fig. 6a). To examine the role of HEY in HCC growth, we subcutaneously inoculated 1 × 10^6^ Huh7-NTC and –shHEY1 cells into BALB/c nude mice. Knockdown of HEY1 markedly retarded HCC growth in vivo (Fig. 6b). We also performed orthotopic implantation with luciferase-labelled MHCC97L-NTC and –shHEY1 cells in BALB/c nude mice. Consistent with the subcutaneous injection model, knockdown of HEY1 reduced the size of orthotopic HCC tumors and impeded pulmonary metastases (Fig. 6c, d). 100% (6/6) of mice injected with MHCC97L-NTC cells showed lung metastasis versus 0% (0/6) of mice injected with MHCC97L-shHEY1 showed lung metastasis (Fig. 6c). Reversely, HEY1 over-expressing MHCC97L cells grew faster than the control cells in orthotopic HCC model (Fig. 6e). We further performed subcutaneous injection with Huh7-NTC and –shPINK1 cells in BALB/c nude mice. Knockdown of PINK1 promoted growth of Huh7-derived subcutaneous tumors, suggesting the suppressive roles of PINK1 in HCC growth (Fig. 6f).

## Discussion

**Fig. 7:**
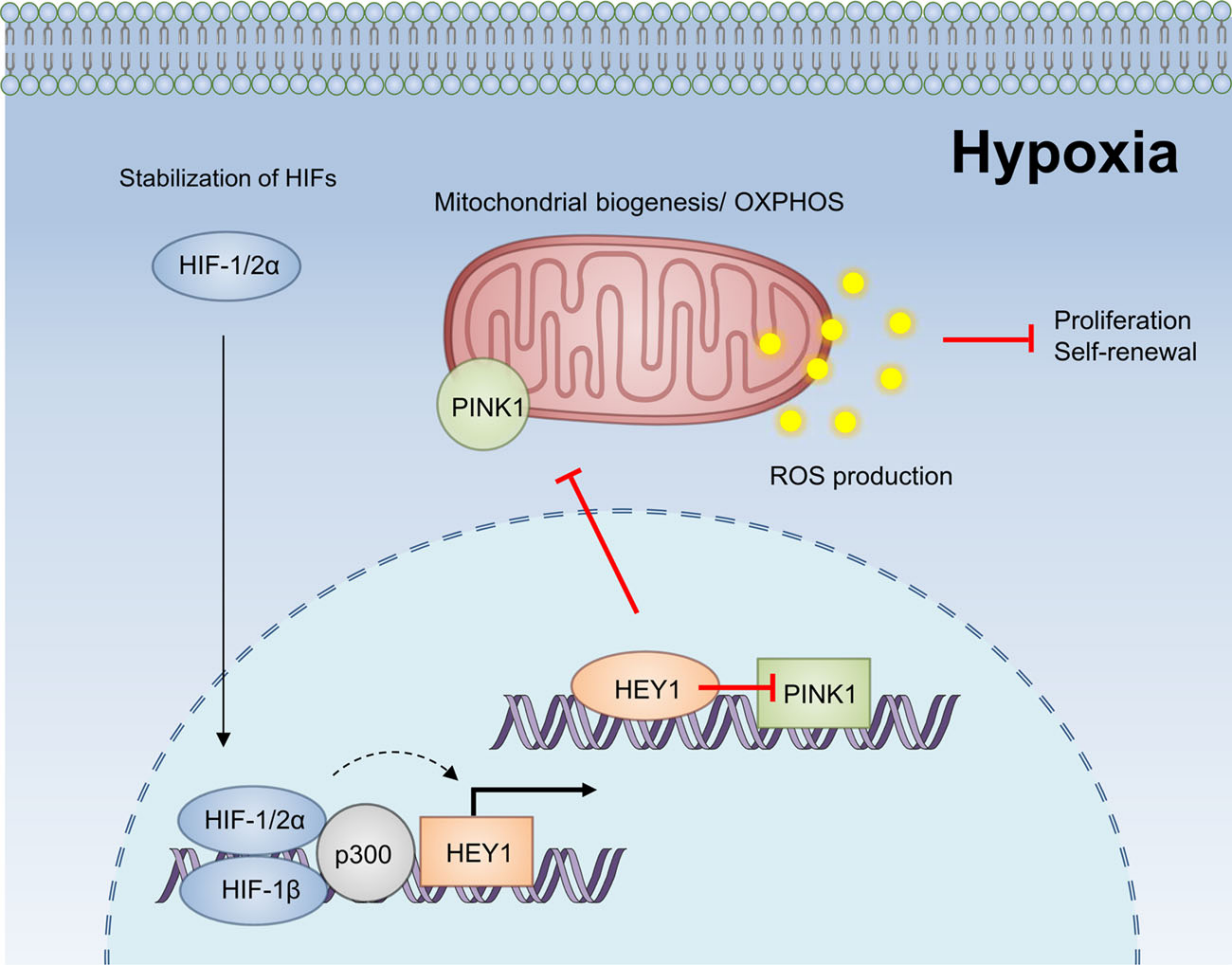
Summary. HIF-1 transcriptionally activates HEY1. HEY1 in turn transcriptionally represses PINK1. PINK1 is important to mitochondrial biogenesis. During hypoxia, ROS accumulates at the mitochondria due to inefficient electron transfer through the electron transport chain in the mitochondria, HIF-1 overcomes the oxidative stress through elevating HEY1 to reduce PINK1-mediated mitochondrial biogenesis, making HCC cells less dependent on mitochondria to reduce mitochondrial ROS production. Therefore, the HIF-1/HEY1 pathway confers survival advantages to HCC cells which frequently experience hypoxia.

Our study provided evidence that hypoxia stabilized HIF-1 which activated the transcription of HEY1 which in turn transcriptionally repressed PINK1 in HCC cells (Fig. 7). We further demonstrated that PINK1 was important to biogenesis of mitochondria, major ROS-producing sites (Fig. 7). HIF-1/HEY1 pathway was able to alleviate ROS production through repressing PINK1-associated mitochondrial biogenesis. We showed that HEY1 was significantly over-expressed in HCC, ICC, and renal cancer while PINK1 was significantly under-expressed in these three cancer types in which HIF was often activated. Up-regulation of HEY1 and down-regulation of PINK1 were tightly correlated. HCC patients with up-regulation of HEY1 or down-regulation of PINK1 had shorter survival rate, suggesting that HEY1 or PINK1 expression might serve as prognostic indicator for HCC. We further found that high HEY1 expression was associated with more advanced HCC stages and low PINK1 expression was associated with poorer cellular differentiation in HCC. Our study has highlighted the clinicopathological significance of HEY1 and PINK1 expression and suggested new prognostic markers for HCC. Furthermore, we showed that activation of HIF-1/HEY pathway counteracted oxidative stress in HCC cells. Whether ROS level is low in HCC tissues with high HEY1 expression could be further studied in the future. Elevation of oxidative stress sensitized HCC to Sorafenib treatment^28–30^. Therefore, at the therapeutic level, HCC with high HEY1 expression are expected to be more resistant to Sorafenib treatment due to the reduced oxidative stress level as compared to HCC with low HEY1 expression.

HEY1 is a transcriptional repressor and its roles in stem cell maintenance have been extensively reported. Here, we reported a new function of HEY1 in mitochondrial biogenesis through PINK1 inhibition. We confirmed that knockdown of PINK1 increased tumor sphere formation, suggesting that HEY1 may confer cancer cell stemness properties through PINK1. From our analysis, there are other interesting HEY1 potential targets which merit further investigation in the future. For example, HES4 is a less studied NOTCH family member which also functions as a transcriptional repressor. HES4 induces the expressions of RUNX2, osteocalcin, osteopontin, and bone sialoprotein in bone marrow stem cells to promote osteogenesis^31^. It is possible that different NOTCH family members cross-talk to control the transcriptional program of cancer cells. ChIP sequencing of HEY1 and different NOTCH family members will provide valuable information with regards to their common and distinct transcriptional targets. Another potential HEY1 target is PIK3CD which encodes for p110δ, an important component of a heterodimeric lipid kinase complex PI3Kδ, which is activated to relay signals when the T and B cell receptors are engaged with antigens^32^. PI3Kδ is crucial to leukocyte and is a central regulator of signaling in B cell malignancies^33^. PI3Kδ, like other PI3K family members, is also activated by receptor tyrosine kinases and controls the Akt/mTOR pathway. Mutations of PI3KCD resulted in immunodeficiency in mice and human^32^. The roles of PI3Kδ in HCC development and its interaction with HEY1 remain to be explored.

Mitochondrion is an important organelle that controls the metabolic flux of cell and is essential to cell survival. Mitochondria are sites where ATP and ROS are produced and therefore need to be tightly controlled for quick adaptations to different environmental conditions or stresses. PINK1 controls mitochondrial biogenesis and safeguards mitochondrial quality in normal cells. PINK1 mutations often drive neurological disorder like the Parkinson’s disease (PD) due to the impairment of normal mitochondrial functions^34,35^. PINK1 maintains the healthy mitochondria and facilitates the degradation of damaged mitochondria. In healthy mitochondria, PINK1 phosphorylates NDUFA10 in the complex I of the ETC to optimize mitochondrial activity and ATP production^36^. PINK1 also phosphorylates BCL-XL and HTRA2 to protect cells from apoptosis^37,38^. PINK1 also protects cells from calcium-induced cell death through phosphorylating unknown targets^39^. In damaged mitochondria, PINK1 self-phosphorylates and phosphorylates PARKIN, DRP1, MFN2, MICRO, ubiquitin, to mediate mitochondrial fission, arrest, and degradation^40–43^. Mitochondria undergo fission to facilitate the removal of damaged mitochondria. Mitochondrial fission involves the division of a mitochondrion into two mitochondria. The abnormal daughter mitochondrion is either cleared by mitophagy/ autophagy or can be fused with a healthy mitochondrion to allow mitochondrial DNA repair, sharing of intact mitochondrial proteins, and restoration of membrane potential and mitochondrial function. The clearance of damaged mitochondria has been shown to be mediated by PINK1^41^. Mitochondrial fission is associated with greater dependence on glycolysis due to fragmented mitochondria while mitochondrial fusion is associated with greater metabolic dependence on oxidative phosphorylation which takes place in mitochondria. Our data showed that hypoxia increases the number and decreases the size of mitochondria, signs of mitochondrial fission, despite PINK1 expression is reduced under hypoxic condition. This observation is coherent with the fact that hypoxic cells have reduced reliance on mitochondria and increased reliance on glycolysis for energy production. This also suggested that reduction of PINK1 is not sufficient to prevent mitochondrial fission. In HCC cells, we saw that knockdown of PINK1 disintegrated the cristae structures of mitochondria instead of impeding mitochondrial fission. Mitochondria could not be found in many PINK1 knockdown HCC cells, suggesting that PINK1 is important to the biogenesis of mitochondria in HCC. It is possible that PINK1 only causes mitochondrial fission in cells with damaged mitochondria which is not the case in our cell model.

Hypoxia elicits ROS accumulation due to the insufficient amount of electron recipient, O_2_, causing imbalanced electron transfer in the ETC. HIF-1 allows hypoxic cells to survive by inducing glycolytic genes, preventing entrance of pyruvate into TCA cycle, and shifting the use of less active ETC components to maintain cells at low ROS level. Furthermore, HIF-1 induces BNIP3 to degrade mitochondria during hypoxic stress to eliminate ROS and promote cell survival together with BECLIN1 and ATG5^44^. In line with all these studies, we unexpectedly found that HIF also reduces mitochondrial biogenesis by up-regulating HEY1 to transcriptionally repress PINK1, suggesting HIF-1 is a central regulator which simultaneously orchestrate various genes for metabolic adaptation. The other way around, it has been demonstrated in mouse embryonic fibroblasts and neurons that knockout of PINK1 was able to elicit ROS which stabilized HIF-1α protein, thereby promoting the transcription of glycolytic genes including GLUT1, GLUT3, HK3, and GAPDH, as well as PDK1 to reprogram cells towards glycolysis^45^. Integrating this and our current studies, it is plausible that HIF-1/HEY1 pathway-induced PINK inhibition might positively feedback to further stabilize HIF-1α and reinforce the pathway. Interestingly, demonstrated in a drosophila model, PINK1 mutation-induced mitochondrial defect could induce a compensatory mechanism involving nucleotide salvage for mitochondrial biogenesis^46^. Whether reduction of PINK1 by HIF-1/HEY1 likewise could elicit compensatory metabolic mechanisms needs to be addressed by metabolomics study in the future.

Another worth-mentioning information is that PINK1 is situated at chromosome 1p36, a frequently deleted region in multiple cancers including HCC^47^. Loss of PINK1 may provide cancer cells metabolic advantages due to reduction of mitochondria-associated oxidative stress. Reduced PINK1 expression and heterozygous mutations of PINK1 were found in glioblastoma^48,49^. Our current study showed that PINK1 expression can be altered as an adaption to change of O_2_ content. Tumor suppressors PTEN and FOX3A have been shown to induce PINK1 and PARKIN^50,51^. In addition to genetic alterations, HIF-1/HEY pathway further represses PINK1 to reduce mitochondrial biogenesis. Strikingly, we found that up-regulation of HEY1 and down-regulation of PINK both have significant prognostic implications and are closely correlated in human HCC samples, further consolidating the importance of this pathway in cancer. Our current work has revealed unexpected role of HEY1 in mitochondrial biogenesis apart from the well-established role of HEY1 in stemness maintenance. Similar to HIF-1, roles of HEY1 could be versatile and more exciting findings could be stemmed from this study. Furthermore, the roles of PINK1 in neuroscience have been established and our study revealed the tumor-suppressive roles of PINK1 in HCC. As PINK1 is also under-expressed in a HIF- dependent cancer type, renal cancer, we believe that our finding is not restricted to HCC but a universal metabolic adaption response that is beneficial to tumor growth.

## Supplementary information

Supplementary Fig. S1. Knockdown and knockout efficiencies of HIF-1α and HIF-2α knockdown or knockout HCC stable cells.

Supplementary Fig. S2. Knockdown efficiencies of HEY1 knockdown HCC stable cells.

Supplementary Fig. S3. HEY1 and PINK1 expressions in kidney (renal) cancer.

Supplementary Fig. S4. PINK1 expression in PINK1 knockdown HCC cells.

Supplementary Fig. S5. PINK1 over-expressing HCC cells have increased mitochondrial mass.

Supplementary Fig. S6.

shRNA and sgRNA sequences.

Primer sequences.

Clinicopathological Correlation of HEY1 in human HCC.

Common genes regulated by HEY1.

Clinicopathological Correlation of PINK1 in human HCC

Clinicopathological Correlation of PINK1 in human HCC.
