# Supplementary figures and images for: Hypoxia regulates the mitochondrial activity of hepatocellular carcinoma cells through HIF/HEY1/PINK1 pathway
Source: Cell Death Dis. 2019 Dec 9;10(12):934. doi: 10.1038/s41419-019-2155-3 (PMC6901483; doi:10.1038/s41419-019-2155-3)

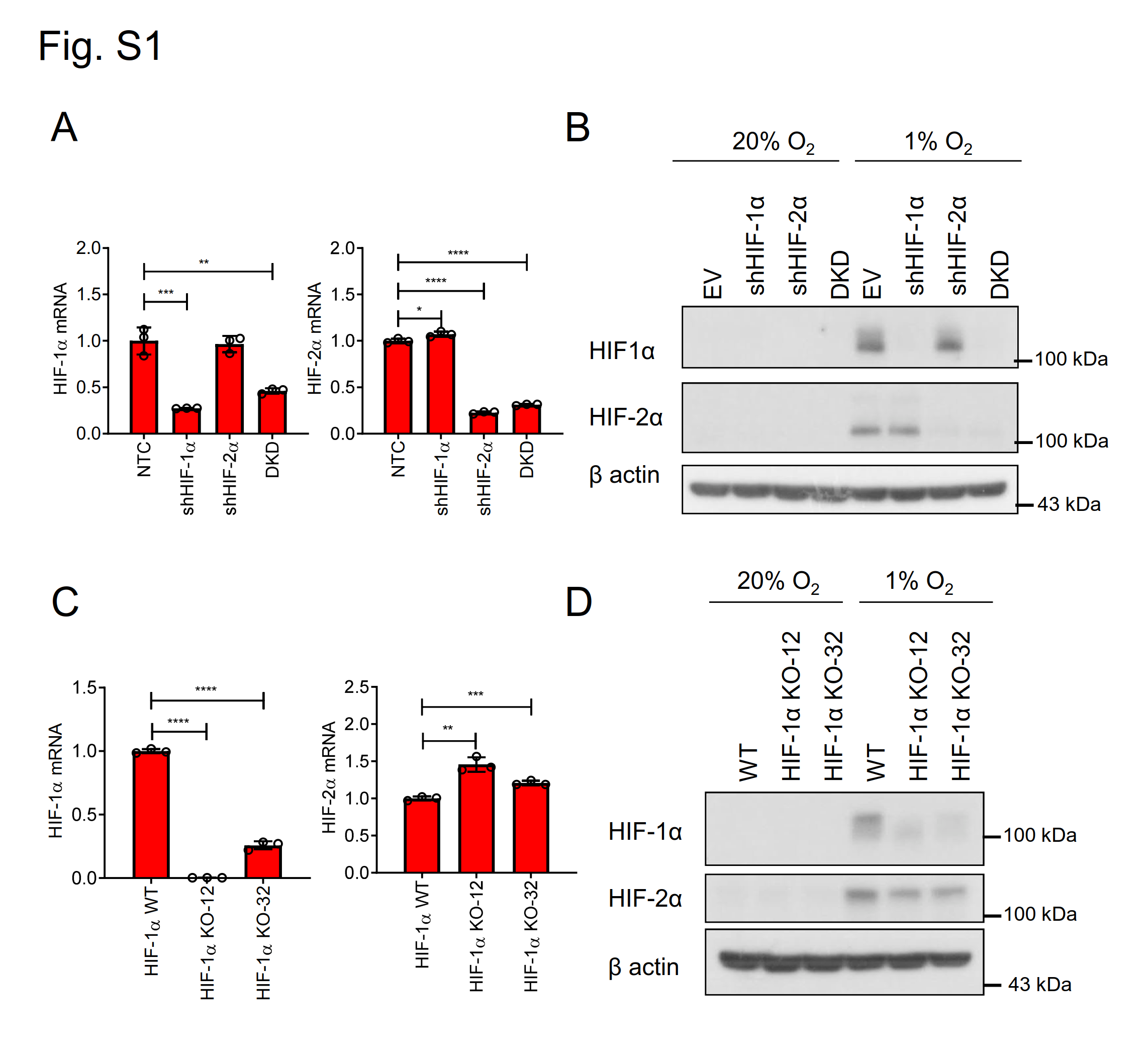

Supplement: Supplementary file 1 — Supplementary Fig. S1. Knockdown and knockout efficiencies of HIF-1α and HIF-2α knockdown or knockout HCC stable cells. [file 41419_2019_2155_MOESM1_ESM.tif]

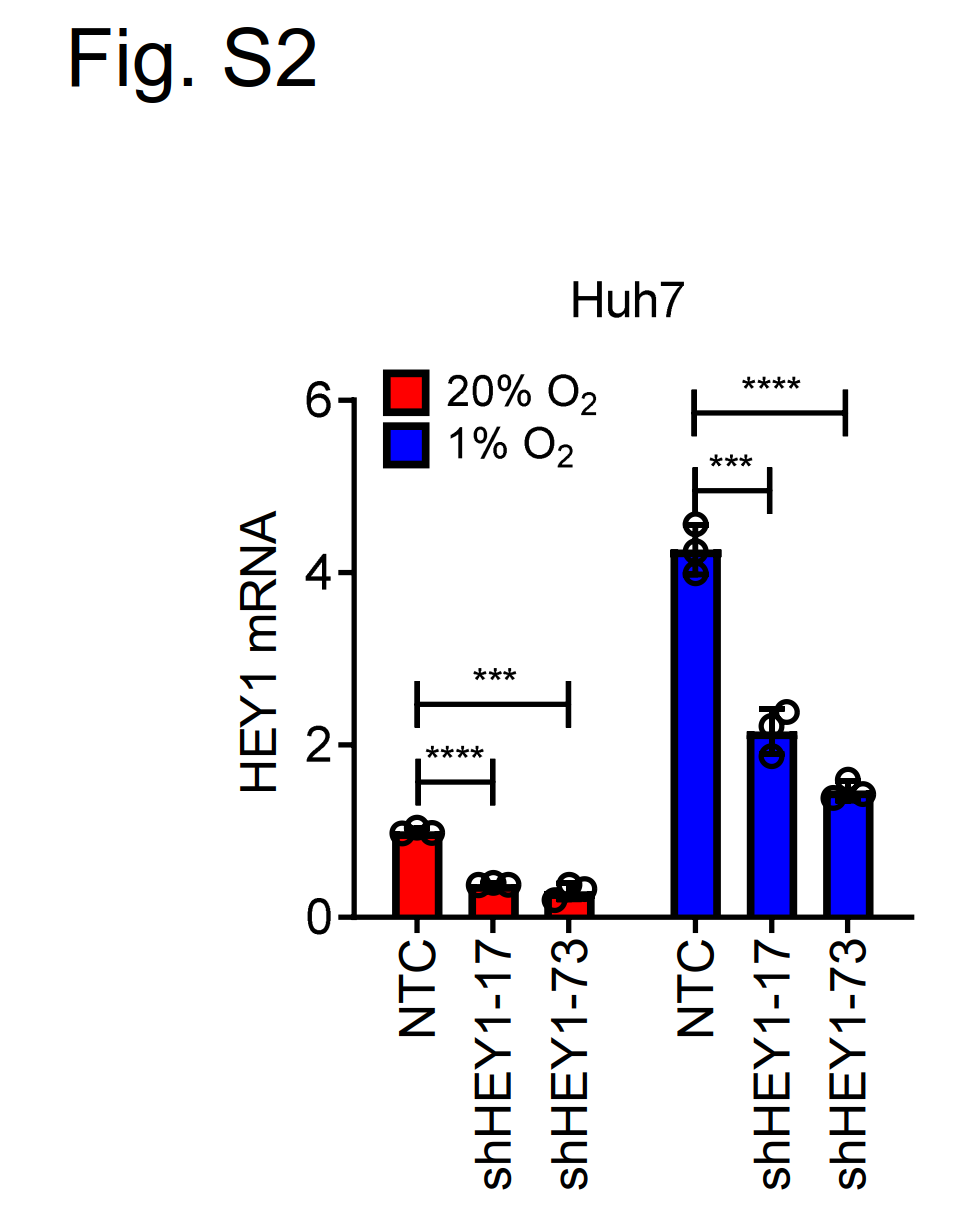

Supplement: Supplementary file 2 — Supplementary Fig. S2. Knockdown efficiencies of HEY1 knockdown HCC stable cells. [file 41419_2019_2155_MOESM2_ESM.tif]

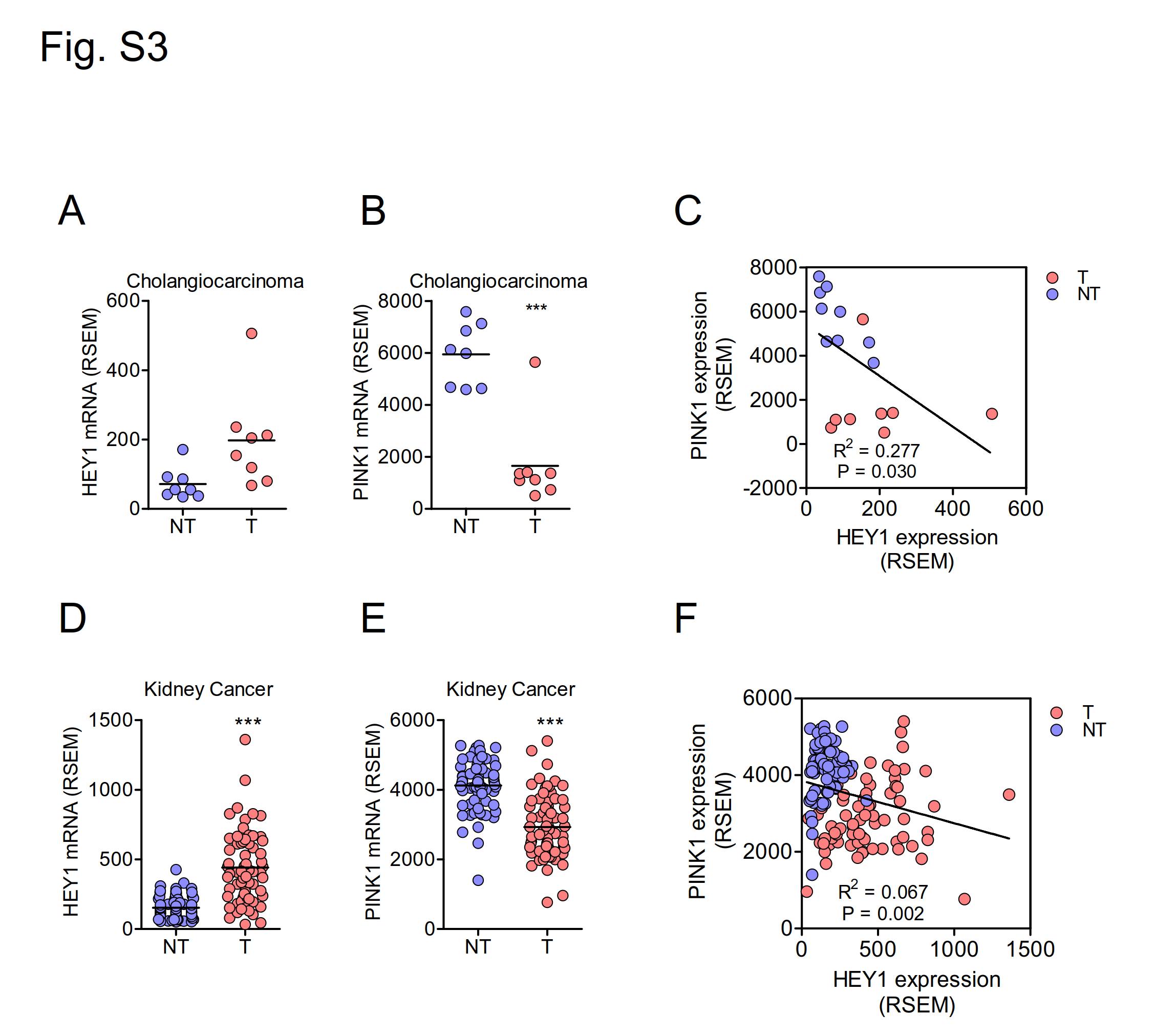

Supplement: Supplementary file 3 — Supplementary Fig. S3. HEY1 and PINK1 expressions in kidney (renal) cancer. [file 41419_2019_2155_MOESM3_ESM.tif]

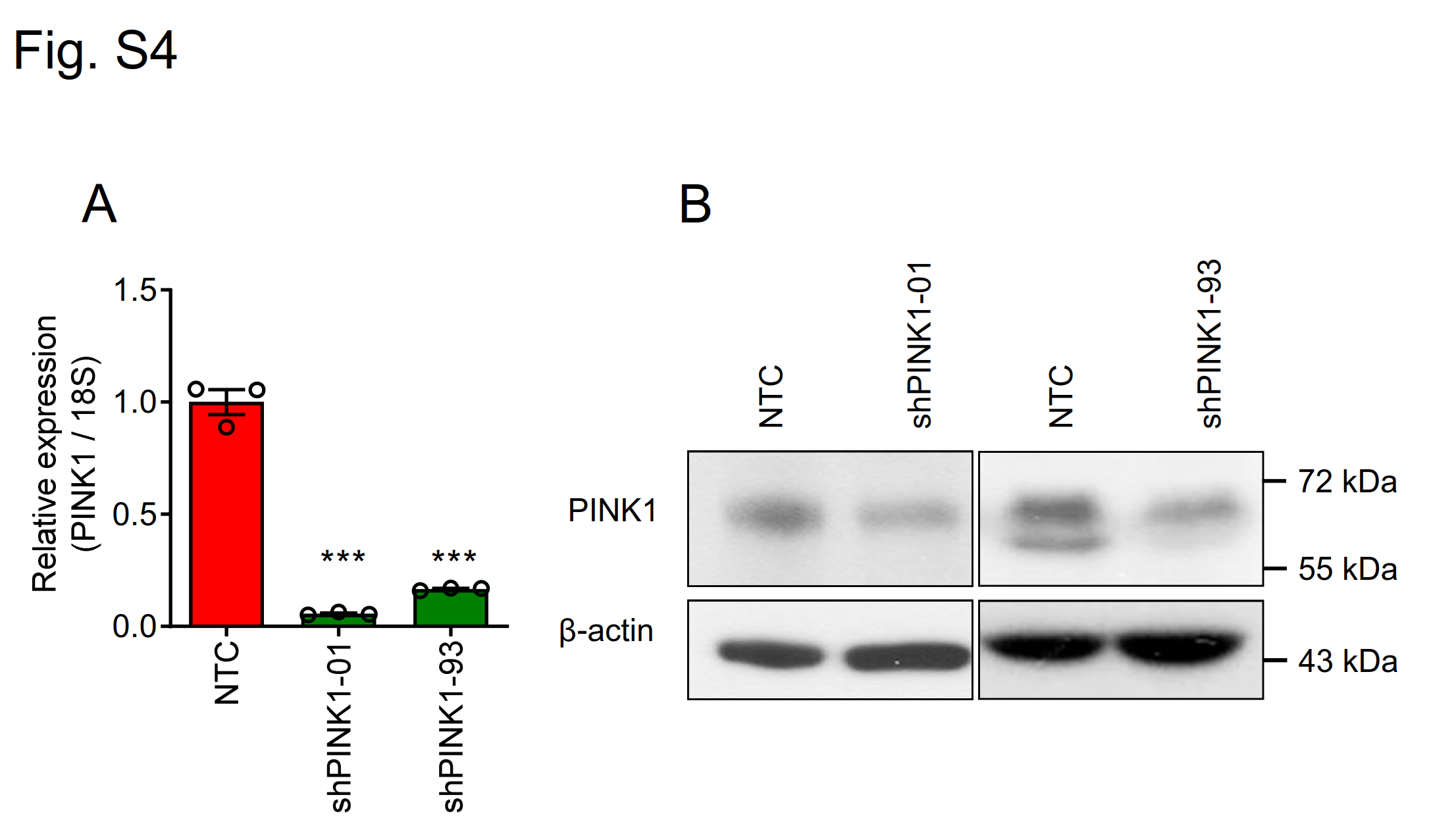

Supplement: Supplementary file 4 — Supplementary Fig. S4. PINK1 expression in PINK1 knockdown HCC cells. [file 41419_2019_2155_MOESM4_ESM.tif]

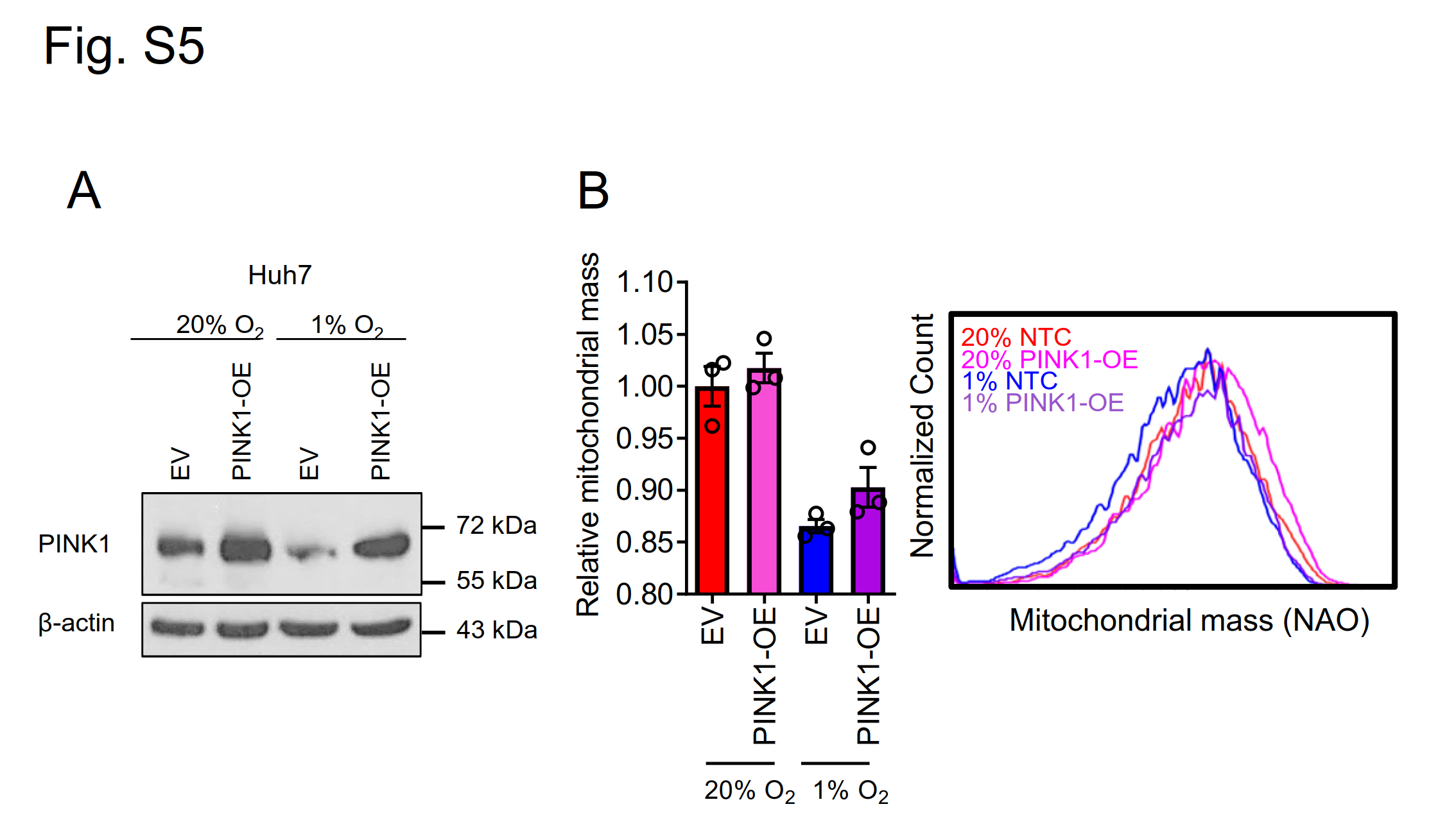

Supplement: Supplementary file 5 — Supplementary Fig. S5. PINK1 over-expressing HCC cells have increased mitochondrial mass. [file 41419_2019_2155_MOESM5_ESM.tif]

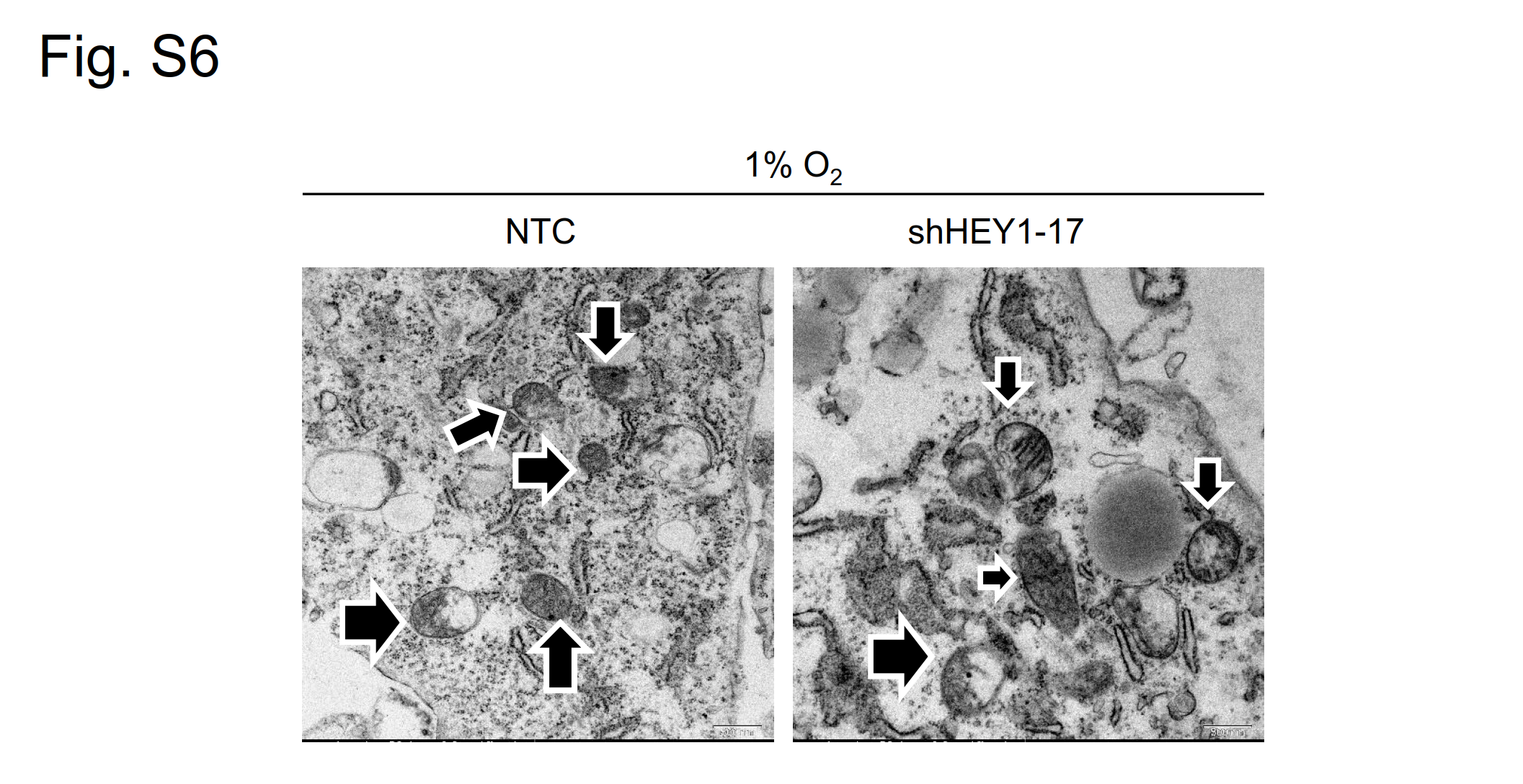

Supplement: Supplementary file 6 — Supplementary Fig. S6. [file 41419_2019_2155_MOESM6_ESM.tif]
