# Supplementary material for: Hypoxia regulates the mitochondrial activity of hepatocellular carcinoma cells through HIF/HEY1/PINK1 pathway
Source: Cell Death Dis. 2019 Dec 9;10(12):934. doi: 10.1038/s41419-019-2155-3 (PMC6901483; doi:10.1038/s41419-019-2155-3)
Supplement: Supplementary file 7 — shRNA and sgRNA sequences. [file 41419_2019_2155_MOESM7_ESM.docx]

**Supplementary Table 1. shRNA and sgRNA sequences.**

| **shRNA/sgRNA clones** | **5’-3’ target sequences** |
| --- | --- |
| sgPINK1-Forward  sgPINK1-Reverse  sgHEY1-1-Forward  sgHEY1-1-Reverse  sgHEY1-3 Forward | AAAGGGAAAGTCACTGCTAG  CTAGCAGTGACTTTCCCTTT  CCGACGCACCGCCGCCGGGC  GCCCGGCGGCGGTGCGTCGG  GCGCGGTCCGCTAGAGCCCT |
| sgHEY1-3 Reverse  shHEY1-17-Forward | AGGGCTCTAGCGGACCGCGC  GCAGGAGGGAAAGGTTACTTT |
| shHEY1-17-Reverse | GCAGGAGGGAAAGGTTACTTT |
| shHEY1-73- Forward | CCGACGAGACCGGATCAATAA |
| shHEY1-73- Reverse | CCGACGAGACCGGATCAATAA |
| shPINK1-01-Forward | CGGCTGGAGGAGTATCTGATA |
| shPINK1-01-Reverse | CGGCTGGAGGAGTATCTGATA |
| shPINK1-93-Forward | CGGACGCTGTTCCTCGTTATG |
| shPINK1-94-Reverse | CGGACGCTGTTCCTCGTTATG |
