# Supplementary material for: Hypoxia regulates the mitochondrial activity of hepatocellular carcinoma cells through HIF/HEY1/PINK1 pathway
Source: Cell Death Dis. 2019 Dec 9;10(12):934. doi: 10.1038/s41419-019-2155-3 (PMC6901483; doi:10.1038/s41419-019-2155-3)
Supplement: Supplementary file 8 — Primer sequences. [file 41419_2019_2155_MOESM8_ESM.docx]

**Supplementary Table 2. Primer sequences.**

| **Primers** | **5’-3’ target sequences** |
| --- | --- |
| BamHI-HEY1-Forward | GGATCCAGAGGCGCCGCTGTAGTTA |
| HEY1-XhoI-Reverse | CTCGAGGGAAAAGCTCCGATCTCCGTCC |
| HEY1-1238-Forward (ChIP) | AAACAACCATTCTGTTTCTGGA |
| HEY1-1238-Reverse (ChIP) | ACACACACCTCTGGCTTCG |
| PINK1-1738-Forward (ChIP) | GTCTTGAACTCCTGGCCTCA |
| PINK1-1738-Reverse (ChIP) | CACAGGCTCACTCATGGAAA |
| PINK1-124-Forward (ChIP) | TCACAAGACCTCGAATGCTG |
| PINK1-124-Reverse (ChIP) | TTTCCCTTTGCACTTTGGAC |
| PINK1-60-Forward (ChIP) | GTCCAAAGTGCAAAGGGAAA |
| PINK1-60-Reverse (ChIP) | CCGGTCACAACAAACTTGG |
| PINK1-Forward | AACATCTCGGCAGGTTCCTC |
| PINK1-Reverse | GCTTGGGACCTCTCTTGGAT |
| HEY1-Forward | GAGTGCGGACGAGAATGGAA |
| HEY1-Reverse | TCGTCGGCGCTTCTCAATTA |
