# Supplementary material for: Hypoxia regulates the mitochondrial activity of hepatocellular carcinoma cells through HIF/HEY1/PINK1 pathway
Source: Cell Death Dis. 2019 Dec 9;10(12):934. doi: 10.1038/s41419-019-2155-3 (PMC6901483; doi:10.1038/s41419-019-2155-3)
Supplement: Supplementary file 9 — Clinicopathological Correlation of HEY1 in human HCC. [file 41419_2019_2155_MOESM9_ESM.docx]

**Supplementary Table 3. Clinicopathological Correlation of HEY1 in human HCC.**

| **Clinicopathological features** | | **HEY1^high^ No. of cases** | **HEY1^low^ No. of cases** | **P Value** |
| --- | --- | --- | --- | --- |
| Sex | Male | 34 | 35 | 0.792 |
|  | Female | 10 | 8 |  |
| Venous invasion | Absent | 24 | 16 | 0.194 |
|  | Present | 20 | 25 |  |
| Tumor encapsulation | Absent | 26 | 28 | 0.500 |
|  | Present | 17 | 13 |  |
| Tumor microsatellite formation | Absent | 24 | 17 | 0.280 |
|  | Present | 20 | 24 |  |
| Hepatitis B surface antigen | Absent | 9 | 10 | 0.798 |
|  | Present | 33 | 31 |  |
| Direct liver invasion | Absent | 22 | 25 | 0.485 |
|  | Present | 17 | 13 |  |
| Hepatitis C surface antigen | Absent | 16 | 16 | 1.000 |
|  | Present | 3 | 3 |  |
| Hepatitis B surface antigen from plasma | Absent | 8 | 7 | 1.000 |
|  | Present | 36 | 36 |  |
| Cellular differentiation by Edmondson grading | I-III | 21 | 19 | 1.000 |
|  | IV-VI | 23 | 22 |  |
| Tumor size | <=5 cm | 19 | 11 | 0.173 |
|  | >5 cm | 25 | 29 |  |
| Cirrhotic liver | Normal/chronic hepatitis | 16 | 22 | 0.130 |
|  | Cirrhosis | 28 | 19 |  |
| Chronic liver disease | Normal | 2 | 4 | 0.423 |
|  | Chronic hepatitis/ cirrhosis | 42 | 37 |  |
| Tumor stage | I/II | 23 | 11 | **0.026*** |
|  | III/IV | 21 | 30 |  |
