# Supplementary material for: Hypoxia regulates the mitochondrial activity of hepatocellular carcinoma cells through HIF/HEY1/PINK1 pathway
Source: Cell Death Dis. 2019 Dec 9;10(12):934. doi: 10.1038/s41419-019-2155-3 (PMC6901483; doi:10.1038/s41419-019-2155-3)
Supplement: Supplementary file 10 — Common genes regulated by HEY1. [file 41419_2019_2155_MOESM10_ESM.docx]

**Supplementary Table 4. Common genes regulated by HEY1.**

| **Gene abbreviation** | **Full name** |
| --- | --- |
| SPSB1 | SplA/Ryanodine Receptor Domain And SOCS Box Containing 1 |
| EFHD2 | EF-Hand Domain Family Member D2 |
| HES4 | Hes Family BHLH Transcription Factor 4 |
| PIK3CD | Phosphatidylinositol-4,5-Bisphosphate 3-Kinase Catalytic Subunit Delta |
| PINK1 | PTEN Induced Putative Kinase 1 |
| CAPZB | Capping Actin Protein Of Muscle Z-Line Beta Subunit |
| FBXO2 | F-Box Protein 2 |
| TMEM51 | Transmembrane Protein 51 |
| CAMTA1 | Calmodulin Binding Transcription Activator 1 |
| NBL1 | Neuroblastoma 1, DAN Family BMP Antagonist |
| GNB1 | G Protein Subunit Beta 1 |
| VPS13D | Vacuolar Protein Sorting 13 Homolog D |
