# Supplementary material for: Hypoxia regulates the mitochondrial activity of hepatocellular carcinoma cells through HIF/HEY1/PINK1 pathway
Source: Cell Death Dis. 2019 Dec 9;10(12):934. doi: 10.1038/s41419-019-2155-3 (PMC6901483; doi:10.1038/s41419-019-2155-3)
Supplement: Supplementary file 11 — Clinicopathological Correlation of PINK1 in human HCC [file 41419_2019_2155_MOESM11_ESM.docx]

**Supplementary Table 5. Clinicopathological Correlation of PINK1 in human HCC.**

| **Clinicopathological features** | | **PINK1^high^ No. of cases** | **PINK1^low^ No. of cases** | **P Value** |
| --- | --- | --- | --- | --- |
| Sex | Male | 36 | 34 | 0.782 |
|  | Female | 7 | 8 |  |
| Venous invasion | Absent | 19 | 19 | 1.000 |
|  | Present | 24 | 23 |  |
| Tumor encapsulation | Absent | 28 | 26 | 0.824 |
|  | Present | 15 | 16 |  |
| Tumor microsatellite formation | Absent | 17 | 17 | 1.000 |
|  | Present | 26 | 25 |  |
| Hepatitis B surface antigen | Absent | 7 | 9 | 1.000 |
|  | Present | 35 | 32 |  |
| Direct liver invasion | Absent | 24 | 23 | 1.000 |
|  | Present | 17 | 16 |  |
| Hepatitis C surface antigen | Absent | 17 | 11 | 0.078 |
|  | Present | 1 | 5 |  |
| Hepatitis B surface antigen from plasma | Absent | 5 | 5 | 1.000 |
|  | Present | 38 | 36 |  |
| Cellular differentiation by Edmondson grading | I-III | 15 | 26 | **0.017*** |
|  | IV-VI | 28 | 16 |  |
| Tumor size | <=5 cm | 18 | 18 | 1.000 |
|  | >5 cm | 25 | 23 |  |
| Cirrhotic liver | Normal/chronic hepatitis | 25 | 18 | 0.195 |
|  | Cirrhosis | 18 | 24 |  |
| Chronic liver disease | Normal | 3 | 2 | 1.000 |
|  | Chronic hepatitis/ cirrhosis | 40 | 40 |  |
| Tumor stage | I/II | 13 | 13 | 1.000 |
|  | III/IV | 28 | 27 |  |
