# Supplementary material for: Hypoxia regulates the mitochondrial activity of hepatocellular carcinoma cells through HIF/HEY1/PINK1 pathway
Source: Cell Death Dis. 2019 Dec 9;10(12):934. doi: 10.1038/s41419-019-2155-3 (PMC6901483; doi:10.1038/s41419-019-2155-3)
Supplement: Supplementary file 12 — Clinicopathological Correlation of PINK1 in human HCC. [file 41419_2019_2155_MOESM12_ESM.docx]

**Supplementary Figure Legends**

**Supplementary Fig. S1. Knockdown and knockout efficiencies of HIF-1α and HIF-2α knockdown or knockout HCC stable cells.** (A) HIF-1α and HIF-2α mRNA expression and (B) protein expression in MHCC97L-HIF-1α single knockdown (shHIF-1α), HIF-2α single knockdown (shHIF-2α), and HIF-1α and HIF-1α double knockdown and control (non-target control, NTC) subclones. (C) HIF-1α and HIF-2α mRNA expression and (D) protein expression in MHCC97L-HIF-1α knockout (KO) stable subclones and wildtype (WT) subclones. For mRNA expression, RT-qPCR was performed with primers specific to HIF-1α and HIF-2α and house-keeping gene 18S. For protein expression, Western blotting was performed in HCC cells exposed to 20% and 1% O_2_ for 4 hours. Data are presented as mean±s.d. (Student’s t-test, * *P*<0.05, ** *P*<0.01, *** *P*<0.001, ***P<0.0001)

**Supplementary Fig. S2. Knockdown efficiencies of HEY1 knockdown HCC stable cells.** (A) HEY1 mRNA expression in Huh7-NTC, -shHEY1-17, and –shHEY-73 stable clones exposed to 20% and 1% O_2_ for 24 hours. Data are presented as mean±s.d. (Student’s t-test, * *P*<0.05, ** *P*<0.01, *** *P*<0.001, ***P<0.0001)

**Supplementary Fig. S3. HEY1 and PINK1 expressions in kidney (renal) cancer.** (A) HEY1 mRNA expression in tumorous tissues (T) and non-tumorous tissues (NT) of 9 cholangiocarcinoma patients. (B) PINK1 mRNA expression in tumorous tissues (T) and non-tumorous tissues (NT) of 9 cholangiocarcinoma patients. (C) PINK1 and HEY1 expressions were inversely correlated in T and NT tissues from cholangiocarcinoma patients. (D) HEY1 mRNA expression in tumorous tissues (T) and non-tumorous tissues (NT) of 72 renal cancer patients. (E) PINK1 mRNA expression in tumorous tissues (T) and non-tumorous tissues (NT) of 72 renal cancer patients. (F) PINK1 and HEY1 expressions were inversely correlated in T and NT tissues from renal cancer patients. RSEM: RNA-Seq by Expectation-Maximization.

**Supplementary Fig. S4. PINK1 expression in PINK1 knockdown HCC cells.** (A) mRNA and (B) protein levels of PINK1 in Huh7-NTC, shPINK1-01, and –shPINK1-93 subclones were evaluated by RT-qPCR and Western blotting, respectively. For qRT-PCR, values were normalized to house keeping gene, 18S. Data are presented as mean±s.d. (Student’s t-test, * *P*<0.05, ** *P*<0.01, *** *P*<0.001)

**Supplementary Fig. S5. PINK1 over-expressing HCC cells have increased mitochondrial mass.** (A) Huh7-EV and –PINK1-OE cells were exposed to 20% and 1% O2 for 48 hours in the presence of FCCP. PINK1 and βactin protein expressions were evaluated by Western blotting. (B) Mitochondrial mass of Huh7-EV and –PINK1-OE cells exposed to 20% and 1% O2 for 24 hours. Data are presented as mean±s.d. (Student’s t-test, * *P*<0.05, ** *P*<0.01, *** *P*<0.001)

**Supplementary Fig. S6.** Mitochondrial structures in HEY1 knockdown HCC cells. Huh7-NTC and –shHEY1-17 cells were cultured in 1% O2 for 24 hours and subjected for TEM imaging.
